# SOX7 Target Genes and Their Contribution to Its Tumor Suppressive Function

**DOI:** 10.3390/ijms19051451

**Published:** 2018-05-14

**Authors:** Yumeng Zhang, Daniel B. Stovall, Meimei Wan, Qiang Zhang, Jeff W. Chou, Dangdang Li, Guangchao Sui

**Affiliations:** 1 College of Life Science, Northeast Forestry University, Harbin 150040, Heilongjiang, China; clover_1211@nefu.edu.cn (Y.Z.); lidd@nefu.edu.cn (D.L.); 2 Division of Math and Science, North Carolina Wesleyan College, Rocky Mount, NC 27804, USA; dstovall@ncwc.edu; 3 Department of Cancer Biology and Comprehensive Cancer Center, Wake Forest University School of Medicine, Winston-Salem, NC 27157, USA; mwan@wakehealth.edu (M.W.); bioqiang@aliyun.com (Q.Z.); jchou@wakehealth.edu (J.W.C.)

**Keywords:** SOX7, gene expression array, breast cancer, target genes, cell proliferation

## Abstract

SOX7 is a transcription factor and acts as a tumor suppressor, but its target genes in cancers are poorly explored. We revealed SOX7-mediated gene expression profile in breast cancer cells using microarray chips and discovered multiple altered signaling pathways. When combinatorially analyzing the microarray data with a gene array dataset from 759 breast cancer patients, we identified four genes as potential targets of SOX7 and validated them by quantitative PCR and chromatin immunoprecipitation assays. Among these four genes, we determined that SOX7-activated *SPRY1* and *SLIT2*, and SOX7-repressed *TRIB3* and *MTHFD2* could all differentially contribute to SOX7-mediated tumor suppression. Overall, we identified multiple cancer-related pathways mediated by SOX7 and for the first time revealed SOX7-regulated target genes in a cancer-relevant context.

## 1. Introduction

SOX7 is a member of the SOX (SRY-related high mobility group (HMG) box) family of transcription factors that are well-characterized regulators of cell fate decisions during development [1]. The SOX family includes 20 members, further grouped into eight (A to H) subfamilies based on their protein similarity. SOX7 belongs to the SOX F subfamily, together with SOX17 and SOX18, and is ubiquitously expressed in normal tissues [2]. SOX7 shares the DNA consensus binding element (5′-(A/T)(A/T)CAA(A/T)G-3′) with many other SOX proteins and has the highest homology (58%) to SOX17. Mice with the homozygous deletion of *SOX7′*s exon 2, which covers 79% of its coding region, are embryonically lethal and those with the heterozygous mutation develop congenital diaphragmatic hernia [3]. To date, only a handful of studies have explored *SOX7* target genes, but none were conducted in a cancer-relevant context; SOX7 activates expression of fibroblast growth factor 3 (*FGF3*) [4], GATA binding protein 4 and 6 (*GATA4* and *GATA6*) [5], laminin alpha 1 (*LAMA1*) [6], vascular endothelial cadherin (*VE-cadherin*, or *CDH5*) [7] and *Notch1* [8]. SOX7 was shown to inhibit expression of RUNX1 target genes in hemogenic endothelium through hindering RUNX1-DNA association [9]. Although the mechanisms of SOX7-mediated tumor suppression are mostly unclear in cancer cells, one study indicated that SOX7 interacted with β-catenin to promote its depletion and consequently inhibited its mediated transcription [10]. SOX7 could inhibit β-catenin-mediated transcription through disrupting the β-catenin/BCL9 interaction [11].

The *SOX7* gene is located on the p arm of human chromosome 8 at a locus frequently deleted in cancers; however, SOX7 homozygous deletions are rare in cancers based on available *SOX7*-related literature. *SOX7* downregulation has been reported for colon, prostate, lung, breast cancers, and myelodysplastic syndrome (a typical prelude to acute myeloid leukemia) [2,10,12,13,14,15,16,17]. Our study and those conducted by others, demonstrated that promoter DNA methylation played a major role for SOX7 downregulation in colon, prostate, breast cancers, and myelodysplastic syndrome [10,15,17]. The 3′-UTR of human SOX7 mRNA has 1973 nucleotides (nts), markedly longer than the average 3′-UTR length (740 nts) of eukaryotic mRNAs [18], implicating its vulnerability as a target of microRNAs. Consistently, many microRNAs have been reported to inhibit SOX7 mRNA [19,20,21,22,23,24]. In addition, *SOX7* is inhibited by a long noncoding RNA that plays an oncogenic role in human glioma [25]. Thus, previous studies strongly suggested a tumor suppressive role of SOX7. Consistently, low SOX7 expression correlated with poor prognoses in patients with lung cancer and myelodysplastic syndrome [12,15]. Our group revealed that *SOX7* downregulation negatively correlated with distant metastasis-free survival in a gene array of 674 breast cancer patients [17].

SOX7 functional studies, although very limited, also suggest a tumor-suppressive role. One of the five reported SOX7-regulated genes is *CDH5* [7], a regulator in epithelial-mesenchymal transition. Overexpressed SOX7 inhibited cell proliferation and colony formation of prostate, breast, and colon cancer cells, induced apoptosis in colon cancer cells, and inhibited tumor formation in breast cancer [10,14,17]. However, whether SOX7 as a transcription factor regulates any cancer-related gene or signaling pathway has never been reported, and our evidence to date suggests that a thorough investigation would be timely.

In this study, we used array chips to determine alterations of gene expression in MDA-MB-231 cells with doxycycline (DOX)-induced SOX7 expression, and discovered multiple potential target genes of SOX7 involved in oncogenesis. When combinatorially analyzing a gene array dataset from a breast cancer cohort, we identified SOX7-activated *SPRY1* (sprouty homolog 1) and *SLIT2* (slit guidance ligand 2) genes, and SOX7-repressed *TRIB3* (Tribbles homolog 3) and *MTHFD2* (methylenetetrahydrofolate dehydrogenase (NADP+ dependent) 2) genes, and determined their roles in SOX7-mediated tumor suppression.

## 2. Results

### 2.1. Microarray Analyses of SOX7 Target Genes

**Figure 1:**
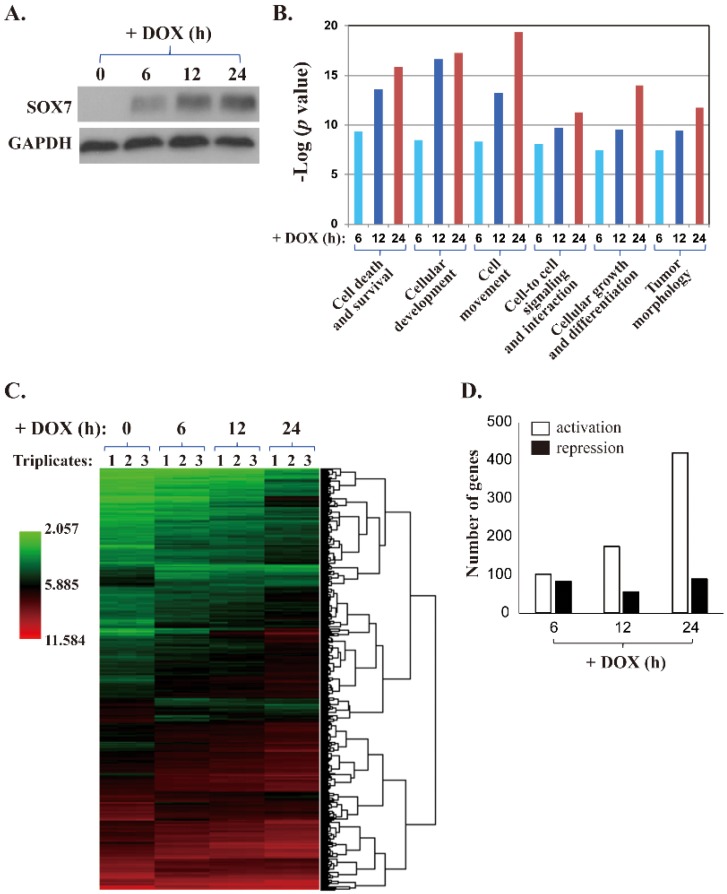
A microarray study of MDA-MB-231 cells with DOX-inducible SOX7 expression. (**A**) A representative Western blot analysis of DOX-induced SOX7 expression in MDA-MB-231 cells. (**B**) In the microarray study, ectopically expressed SOX7 caused significant expression changes in genes of different functional groups. The *p* values are shown as “−log”. For example, the *p* values of the changes at the 3-time points of “Cell Death and Survival” genes are 5.0 × 10^−10^, 2.6 × 10^−14^, and 1.4 × 10^−16^, respectively. The overall *p* values increase from left to right. (**C**) The heat map of the microarray data showing the genes with altered expression between the samples with DOX-induced SOX7 and control. (**D**) Numbers of genes with at least 2-fold of activation or repression in MDA-MB-231 cells with DOX-inducible SOX7 collected at 6, 12 and 24 h after DOX addition.

We reported a tumor suppressive role of SOX7 and its downregulation by DNA methylation in breast cancer [17]. However, the target genes of SOX7 have not been well studied. To characterize the SOX7 target gene profile, we generated a DOX-inducible expression system for SOX7 in MDA-MB-231 cells and collected cells for total RNA extraction at time points of 0, 6, 12 and 24 h after DOX addition to 1.0 μg/mL, which covered early and late responsive genes to SOX7 expression. We validated induced SOX7 expression at these time points by Western blot analysis and observed a steady increase of SOX7 protein (Figure 1A). The collected RNA samples were analyzed by the Affymetrix Human Genome U219 Array strips and the gene expression data at 6, 12 and 24 h time points were normalized against the control (0 h). We detected highly significant changes in the expression of genes from different ontological categories (Figure 1B). Importantly, the genes involved in many essential cancer-related processes, such as cell death, survival, growth, proliferation and tumor morphology, showed remarkable alterations, strongly suggesting that SOX7-mediated gene transcription plays an essential role in oncogenesis. The detailed signaling pathways analyzed by the Kyoto Encyclopedia of Genes and Genomes (i.e., KEGG database) and altered regulatory genes affected by SOX7 induction are shown in Figure S1. The gene expression dataset of this microarray study is available in the Gene Expression Omnibus (GEO) with an access number of GSE111122. Based on the microarray data, we generated a heat map through hierarchical clustering for genes with altered expression in MDA-MB-231 cells with DOX-induced SOX7 (Figure 1C). Overall, based on this heatmap, we observed that most of the affected genes showed monotonically increased expression with SOX7 induction (green to black to red changes, from left to right, Figure 1C), suggesting that more genes were stimulated than repressed. At 6, 12 and 24 h after DOX addition, the numbers of genes upregulated by 2.0 or more folds are 102, 174 and 420, respectively, while the numbers of genes downregulated at least 2.0-fold are 83, 56 and 89, respectively (Figure 1D). The information of these genes, including gene names, NCBI accession numbers, the fold of changes, *p* values, etc., are listed in Table S2. The results that more genes were activated than repressed in our microarray data were consistent with a previous study showing that SOX7 possesses a transactivation domain but lacks any well-defined transrepression domain [26]; consistently, the effects of SOX7 on the expression of its target genes reported in literature are mostly activating [4,5,6,7].

### 2.2. Identifying Essential SOX7 Target Genes in Breast Cancer Development

We discovered many genes with altered expression when SOX7 was inducibly expressed in MDA-MB-231 cells. However, whether these genes are truly clinically relevant to the biological function of SOX7 was unknown. To explore genes potentially regulated by SOX7 in tumor samples, we analyzed the correlation of SOX7 expression with all other genes in a dataset from a cohort of human breast cancer patients (designated as Brca759 dataset) [27] and compared the correlation back to our observations from the microarray (designated as SOX7-U219 dataset). Meanwhile, we evaluated previously reported biological functions of these potential target genes of SOX7.

**Figure 2:**
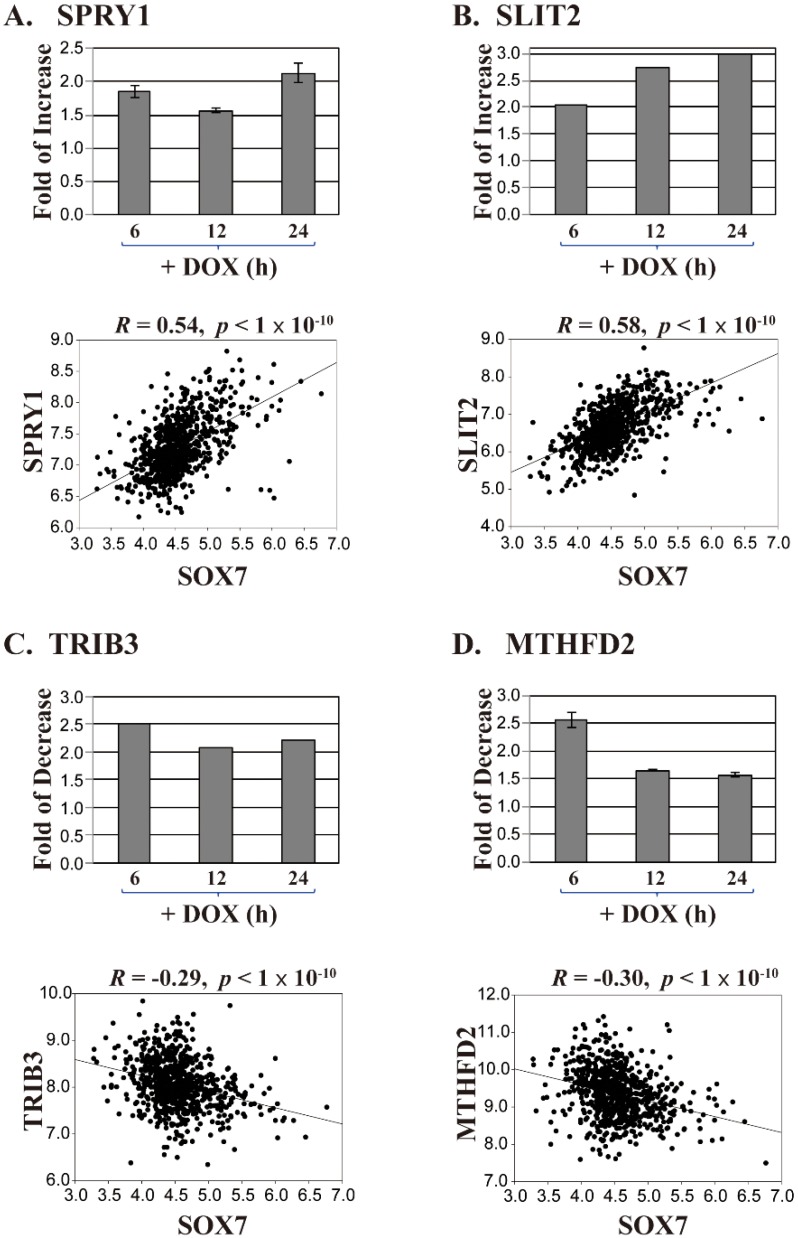
The fold changes and correlation coefficients of four SOX7 potential target genes. (**A**–**D**) Four SOX7 target genes *SPRY1*, *SLIT2*, *TRIB3* and *MTHFD2*, respectively, were selected based on the fold changes at the 3 time points in the SOX7-U219 dataset (upper panels) and the correlation coefficients in the BrCa759 dataset (lower panels; Log2 signal intensity). The coefficients and *p* values for the correlation are labeled.

**Figure 3:**
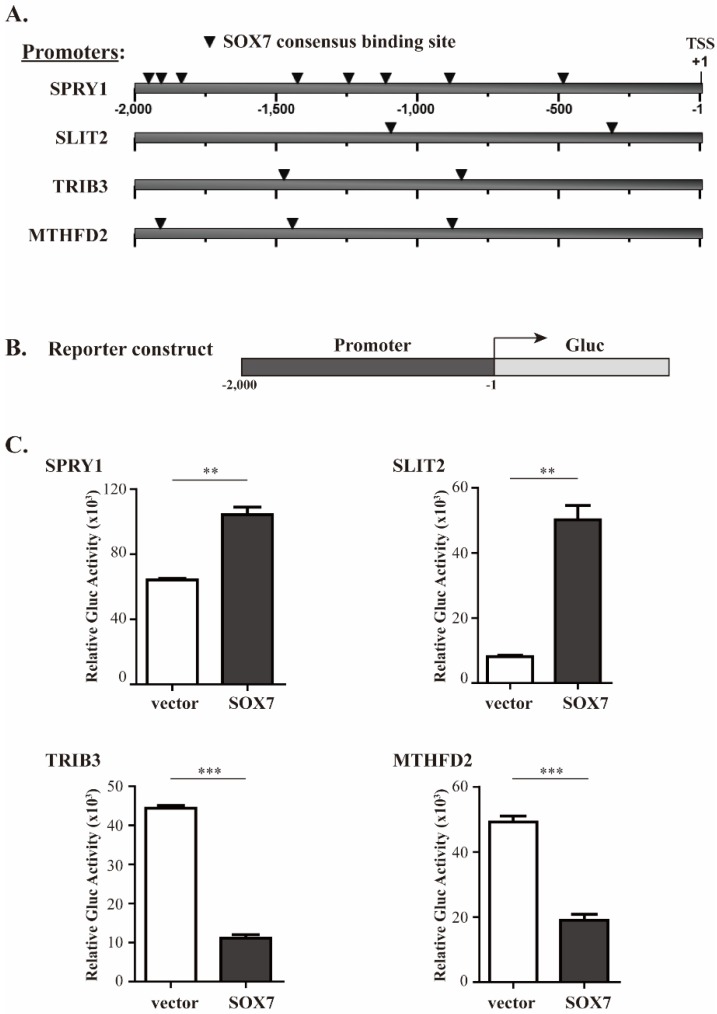
Reporter assays to examine SOX7 effects on the transcription mediated by different promoters. (**A**) Predicted SOX7 consensus sites (pointed by ▼) on the promoters of its potential target genes based on the Tfsitescan [29]. The first nucleotide of a transcription start site (TSS) is designated as “+1”, and the first nucleotide downstream TSS is “−1”. (**B**) Schematic diagram of a reporter construct with a 2 kb promoter driving the expression of Gluc. (**C**) Effects of ectopic SOX7 expression on the Gluc expression driven by the promoters of *SPRY1*, *SLIT2*, *TRIB3* and *MTHFD2*, respectively. ** as *p* < 0.01, *** as *p* < 0.001.

With these abovementioned considerations, we identified four candidate genes; *SPRY1* and *SLIT2* that are likely activated, and *MTHFD2* and *TRIB3* that are repressed by SOX7. In the SOX7-U219 dataset, *SPRY1* and *SLIT2* were both upregulated around or above 2-fold, respectively (Figure 2A,B, upper panels); in the Brca759 dataset, their correlation coefficients to *SOX7* were 0.54 and 0.58, respectively (Figure 2A,B, lower panels, *p* < 1 × 10^−10^), suggesting a positive regulation of *SPRY1* and *SLIT2* gene expression by SOX7. On the other hand, *TRIB3* and *MTHFD2* were downregulated around 2-fold in the SOX7-U219 dataset (Figure 2C,D, upper panels), and they showed significantly inverse correlations to SOX7 with correlation coefficients of −0.29 and −0.30, respectively (Figure 2C,D, lower panels, *p* < 1 × 10^−10^), suggesting a negative regulation of *TRIB3* and *MTHFD2* gene expression by SOX7. To further investigate the potential of these four genes regulated by SOX7, we checked the promoter regions of these four genes. For most eukaryotic genes, the promoter region regulating the gene expression is roughly 1000 bps upstream of its transcription start site (TSS) [28]. To ensure that we would identify functional SOX7 binding sites in the promoters of its potential target genes, we retrieved 2000 bps (or “2 kb”) sequence upstream of the TSS of each SOX7 target gene from the NCBI Genome database. Using the Tfsitescan [29] that predicts transcription factor binding sites, we identified multiple SOX7 consensus sites, i.e., (A/T)(A/T)CAA(A/T)G [30,31], in the positive or negative strands of the 2 kb promoter regions of *SPRY1*, *SLIT2*, *MTHFD2* and *TRIB3* (Figure 3A and Table S3 for the detailed sequences of the binding sites in the four promoters). Collectively, these data strongly suggest that SOX7 activates *SPRY1* and *SLIT2*, but inhibits *MTHFD2* and *TRIB3* in breast cancer.

### 2.3. Validation of SOX7 Regulation of Its Four Target Genes

To determine whether SOX7 regulates transcriptional activity driven by the promoters of *SPRY1*, *SLIT2*, *MTHFD2* and *TRIB3*, we amplified their 2 kb promoter regions and subcloned each promoter upstream of the Gluc coding sequence to generate four reporter constructs (Figure 3B). We then cotransfected each reporter construct, *pcDNA3-SOX7* or an empty vector, and a control vector *pCMV-SEAP* into HeLa cells. The normalized Gluc activity against SEAP activity shown in Figure 3C indicated that SOX7 stimulated the activity of the *SPRY1* and *SLIT2* promoters by 1.6- and 6.2-fold, respectively. On the contrary, SOX7 overexpression could reduce the promoter activity of *TRIB3* and *MTHFD2* by 4.0- and 2.6-fold, respectively. Thus, these data strongly suggested that SOX7 directly regulates the expression of *SPRY1*, *SLIT2*, *TRIB3* and *MTHFD2*.

**Figure 4:**
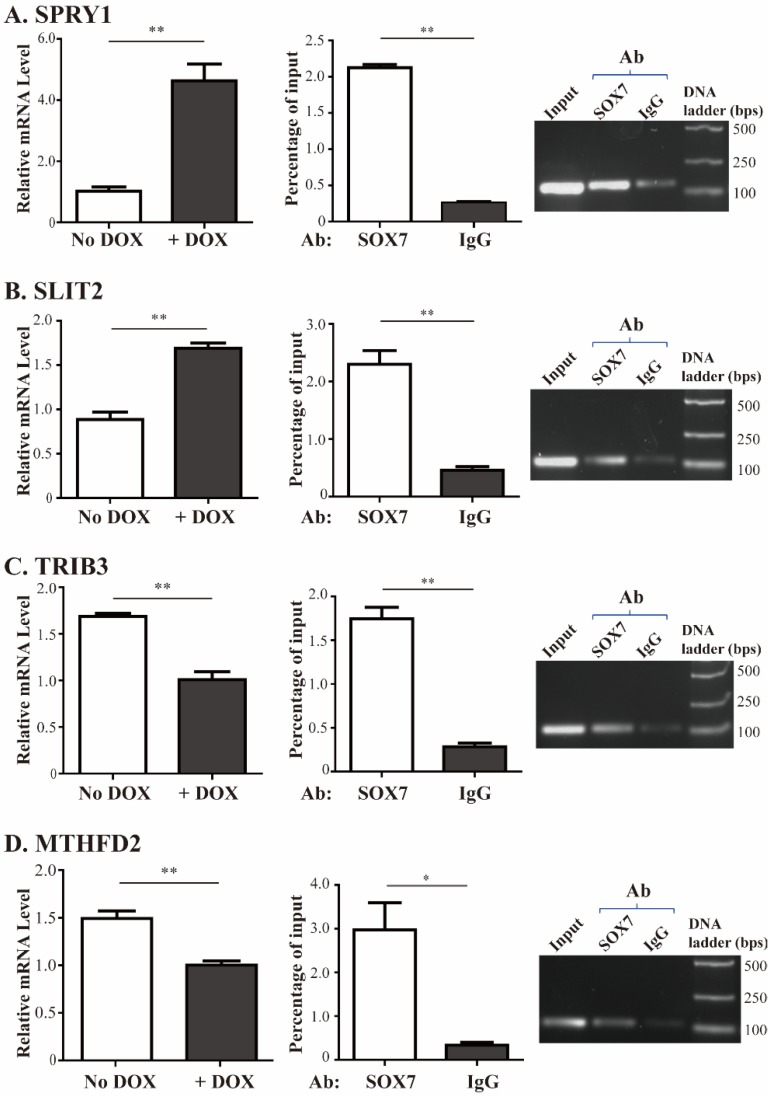
Validation of SOX7-mediated transcription of endogenous target genes. (**A**–**D**): left panels are the quantitative PCR analyses for *SPRY1*, *SLIT2*, *TRIB3*, and *MTHFD2*, respectively, in reversely transcribed RNA samples extracted from MDA-MB-231 cells with DOX-induced SOX7 expression and its control (No DOX). Middle and right panels are the chromatin immunoprecipitation (ChIP) assays for these four genes in MDA-MB-231 cells using the SOX7 antibody and a normal IgG followed by quantitative PCR and semi-quantitative PCR, respectively. Ab: antibody. * as *p* < 0.05, ** as *p* < 0.01.

To validate the results from the SOX7 microarray dataset, we used quantitative RT-PCR to determine the changes of endogenous *SPRY1*, *SLIT2*, *TRIB3* and *MTHFD2* gene expression in MDA-MB-231 cells with DOX-induced SOX7. DOX was added to the medium of MDA-MB-231 cells with DOX-inducible SOX7. After 48 h we collected the cells and extracted total RNA, followed by reverse transcription and quantitative PCR for the four genes. Consistent with the microarray results, SOX7 induction led to increased expression of *SPRY1* and *SLIT2* (by 4.5- and 1.9-fold, respectively, Figure 4A,B, left panels), and reduced expression of *TRIB3* and *MTHFD2* (by 1.7- and 1.5-fold, respectively, Figure 4C,D, left panels). *CDH5* is one of previously identified SOX7 target genes [7], and it was activated by 3.88-fold at 24 h time point after SOX7 induction. We validated its upregulated by RT-qPCR (Figure S2). To determine whether SOX7-mediated gene expression is independent of cell lines, we tested two additional breast cancer cell lines, MDA-MB-453 and MCF-7, and found that the four potential SOX7 target genes identified in MDA-MB-231 cells also showed corresponding up- or downregulation in response to ectopically expressed SOX7 in these two cell lines (Figure S3B,C).

Meanwhile, we carried out ChIP assays to determine whether SOX7 could bind to the promoters of these four genes. As shown in the quantitative and semi-quantitative PCRs in the right two panels of Figure 4A–D, the signal of the promoter fragments of these four genes immunoprecipitated by a SOX7 antibody was significantly higher than that brought down by a control IgG. These results confirmed microarray data that SOX7 directly activated *SPRY1* and *SLIT2*, and repressed *TRIB3* and *MTHFD2* gene expression.

### 2.4. Investigating Contributions of SOX7 Target Genes to Its Tumor Suppressive Role

We next evaluated the contributions of these SOX7 target genes to its tumor suppressive role in breast cancer. The rationale is that if a target gene is essential to SOX7-mediated tumor suppression, reversely changing the expression of this gene will counteract the antiproliferative effects of ectopic SOX7 expression on breast cancer cells.

**Figure 5:**
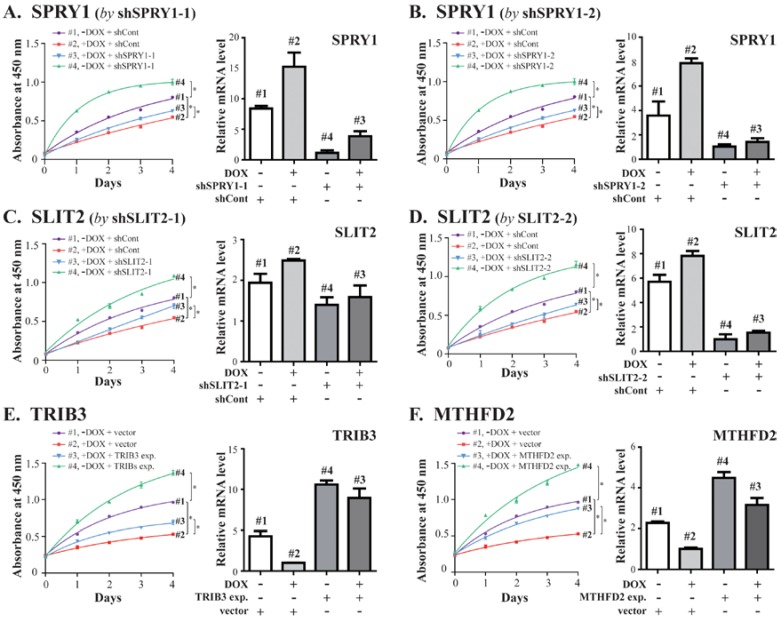
The examination of SOX7 target genes for their roles in SOX7-suppressed cell proliferation. Lentiviruses carrying shRNAs infected MDA-MB-231 cells with DOX-inducible SOX7, ectopically expressed coding sequences, or an empty vector, as labeled, and cultured in the absence or presence of DOX (i.e., −DOX and +DOX, respectively). WST-1 assays determined cell proliferation, and quantitative RT-PCR analyzed gene expression. (**A**,**B**) are the two shRNAs for *SPRY1*; (**C**,**D**) are the two shRNAs for *SLIT2*; (**E**,**F**) are the ectopic expression of *TRIB3* and *MTHFD2*, respectively. * as *p* < 0.05.

Induced SOX7 could upregulate the expression of *SPRY1* and *SLIT2*, so we generated two shRNAs for each of them (shSPRY1-1 and -2, shSLIT2-1 and -2) targeting different sites of their mRNAs, as well as a control shRNA (shCont), and produced lentiviruses carrying these shRNAs. Each of these shRNA lentiviruses individually infected MDA-MB-231 cells with the established DOX-inducible SOX7 system, and the cells were cultured in absence or presence of DOX, followed by WST-1 assays to evaluate cell proliferation, and quantitative RT-PCR to determine gene expression. With induced SOX7 expression (+DOX), MDA-MB-231 cells showed decreased proliferation (compare curves #2 with #1, Figure 5A–D, left panels). In the condition of inducible SOX7 expression (+DOX), simultaneously silencing *SPRY1* or *SLIT2* could partially restore cell proliferation (curves #3 versus #2, Figure 5A–D, left panels). Without SOX7 expression (−DOX), knockdown of either *SPRY1* or *SLIT2* could markedly enhance cell proliferation (curves #4 versus #1, Figure 5A–D, left panels), suggesting the growth inhibitory activity of these two genes. The silencing of *SPRY1* and *SLIT2* by shRNAs were confirmed by quantitative PCR (Figure 5A–D, right panels).

Previous studies suggested the proliferative activity of TRIB3 and MTHFD2 [32,33]. SOX7 negatively regulated and inversely correlated with their expression. Thus, we wanted to determine whether ectopic expression of TRIB3 or MTHFD2 could counteract the tumor suppressive activity of SOX7. We individually amplified the coding regions of *TRIB3* and *MTHFD2* and inserted them into a lentiviral expression vector pSL11 with their expression driven by the chicken β-actin promoter. With inducible SOX7 expression, simultaneously expressed TRIB3 or MTHFD2 could partially restore cell proliferation (curves #3 versus #2, Figure 5E,F, left panels). Without SOX7 expression, ectopically expressed TRIB3 or MTHFD2 could markedly increase cell proliferation, suggesting the growth promoting activity of these two genes (curves #4 versus #1, Figure 5E,F, left panels). The overexpression of *TRIB3* and *MTHFD2* were confirmed by quantitative PCR analyses (Figure 5E,F, right panels).

## 3. Discussion

Human SOX7 was demonstrated as a tumor suppressor in various malignancies, and its downregulation in cancer cells was attributed to promoter DNA methylation and microRNA-mediated inhibition. Regarding the mechanisms underlying SOX7-mediated tumor suppression, it was only proposed that SOX7, like several other SOX proteins, interacts with β-catenin to promote its depletion and inhibit its mediated transcription [10,11,34]. However, we still lacked the knowledge regarding the genes directly regulated by SOX7 to exert its tumor suppressive activity in oncogenic processes. The goal of this study was to fill in this gap. We first carried out the gene chip study in MDA-MB-231 cells with ectopic SOX7 expression, and then selected several candidate genes followed by further investigation of their roles in SOX7-mediated inhibition of cell proliferation.

To choose potential target genes of SOX7 in breast cancer, we took both our SOX7 microarray data and a gene array dataset of 759 breast cancer patients into consideration. We also considered the functional roles of these target genes during oncogenesis. The two selected SOX7-activated genes were previously demonstrated to have antiproliferative activities. SPRY1 inhibits multiple pathways related to oncogenesis, including receptor tyrosine kinase signaling [35], epidermal growth factor-induced proliferation and angiogenesis [36]. It is downregulated in breast cancer and may act as a tumor suppressor [37]. SLIT2 also has tumor suppressive activity and is frequently inactivated in multiple carcinomas, including breast cancer [38]. For the two SOX7-repressed genes, previous studies suggested their proliferative roles in cancer development. TRIB3 binds SMAD3 to promote tumor cell migration and invasion [32] and activates MAPK and TGFβ pathways [33]. Increased TRIB3 could promote acute promyelocytic leukemia by stabilizing PML-RARα and inhibiting p53-mediated senescence [39]. In addition, *TRIB3* overexpression correlated with a poor prognosis of breast cancer patients [40]. MTHFD2 positively regulates vimentin, a key regulator in the epithelial to mesenchymal transition, and promotes cell invasion [41]. Its downregulation reduces cell viability and promotes cell apoptosis [42], while its increased expression correlated with poor prognosis of breast cancer [43]. Overall, our selection of the four SOX7 target genes for further investigation was well justified.

Studies undertaken by our group previously demonstrated that many breast cancer cell lines, including MDA-MB-231, showed undetectable or very low levels of both SOX7 protein and mRNA [17]. The DOX-inducible system could provide a quick increase of ectopic SOX7 in MDA-MB-231 cells to determine SOX7-mediated gene expression. To distinguish genes that were directly and indirectly altered by SOX7, we collected cells for RNA extraction at the three-time points 6, 12 and 24 h after DOX addition. The four genes selected by us showed similar activation or repression in these three samples, suggesting that they are likely to be direct targets of SOX7.

With induced SOX7 expression, silencing of either *SPRY1* or *SLIT2* could only partially restore cell proliferation, suggesting that the growth retardation caused by SOX7 overexpression likely involved an activation of multiple tumor suppressive genes—thus inhibiting one of them would not fully restore cell proliferation. Alternatively, introducing MTHFD2 could markedly rescue cell growth caused by SOX7 overexpression, suggesting that MTHFD2 inhibition played a key role in SOX7-mediated tumor suppression. In contrast, expression of TRIB3 only restored cell growth slightly, implicating that its role in the SOX7 network is less important than MTHFD2. In these experiments, without induced SOX7 expression, knockdown of either *SPRY1* or *SLIT2*, or overexpression of either TRIB3 or MTHFD2 could markedly enhance cell proliferation. These data suggest that the malignancy of MDA-MB-231 cells could be further promoted, with additional oncogenic alterations.

Currently reported effects of SOX7 on its direct target genes are activation, although not studied in a cancer-relevant context [4,5,6,7]. Interestingly, among the five previously identified SOX7 target genes, only *CDH5* was upregulated at the 24 h time point after SOX7 induction in MDA-MB-231 cells. This phenomenon suggested that SOX7 in tumor cells may exert its regulatory role through signaling pathways distinct from these in nontumorigenic scenarios. In the current study, we systematically investigated SOX7-mediated transcription in breast cancer MDA-MB-231 cells, which have an undetectable endogenous SOX7 expression, and discovered *TRIB3* and *MTHFD2* as its repressed genes. Importantly, our data suggested that *MTHFD2* inhibition likely played a key role for SOX7 to exert its antiproliferative activity. Although SOX7 has been recognized as a transcription factor lacking any obvious transrepression domain [26], it is possible that SOX7 may achieve its repressive effects through different mechanisms, such as blocking the entrance of transactivators to the target promoters. Note that in the 2 kb promoter regions, SOX7 has eight potential binding sites in the *SPRY1* promoter, but has two in the *SLIT2* promoter (Figure 3A). However, in response to SOX7 induction, the fold changes of these two genes were not proportional to their binding site numbers. Thus, it is possible that SOX7 did not bind to all potential binding sites in the *SPRY1* promoter, or its association with some of these binding sites exerted a counteractive effect on *SPRY1* gene expression. Future study is needed to dissect differential roles of these eight putative SOX7 binding sites in regulating *SPRY1* gene transcription.

Interestingly, at 6 h after adding DOX to induce SOX7 expression, we detected 102 upregulated genes and 83 downregulated genes by at least 2-fold. However, at the time points of 12 and 24 h, the numbers of upregulated genes dramatically increased, but the numbers of downregulated genes just slightly changed. The genes showing altered expression in the two later time points should contain more indirectly affected genes than the 6 h time point. Thus, SOX7 could likely both activate and repress gene expression. Other SOX proteins also showed repressive activity in mediating gene expression. SOX17, another member of the SOX F subfamily to which SOX7 belongs, repressed *Runx1* and *Gata2* gene expression through directly binding to their promoters in hemogenic endothelial cells [44]. SOX4 and SOX9 could also repress *Cdkn1a* and *α-sarcoglycan* gene expression, respectively [45,46]. Thus, it is not surprising that the transcription factor SOX7 could also repress expression of various genes.

Overall, we have identified an array of potential target genes of SOX7 and examined four of them for their roles in SOX7-mediated tumor suppression. We found that SOX7 could both activate and repress its target genes; and achieve its antiproliferative function through regulating multiple genes simultaneously.

## 4. Materials and Methods

### 4.1. Oligonucleotides, DNA Vectors, and Antibodies

Genewiz (South Plainfield, NJ, USA) was used to synthesize oligonucleotides for PCR and DNA sequencing. The oligonucleotides used in amplifying the promoters of *SPRY1*, *SLIT2*, *TRIB3* and *MTHFD2*, amplifying the coding sequences of *TRIB3*, and *MTHFD2*, conducting quantitative PCR, and generating shRNA constructs (including target site sequences) are listed in Table S1. We used the nest PCR method to amplify the four promoter regions and subcloned them upstream of the Gaussia luciferase (Gluc) coding sequence to generate reporter constructs. The coding sequences of *TRIB3* and *MTHFD2* were also amplified by nest PCR method and subcloned into a lentiviral vector pSL11 with the chicken *β-actin* promoter driving their expression. The construction of shRNAs for *SPRY1* and *SLIT2* followed a procedure described by us previously [47,48]. The inducible expression system was previously reported by us [17]. The antibodies against SOX7 (AF2766; R&D Systems, Minneapolis, MN, USA) and GAPDH (10R-G109A, Fitzgerald Industries International, Acton, MA, USA) were used in Western blot studies.

### 4.2. Cell Culture, Lentiviral Production and Infection

Human breast cancer MDA-MB-231 cells were cultured in RPMI medium containing 10% fetal bovine serum (FBS) and 1% penicillin-streptomycin and maintained at 37 °C in an atmosphere of 5% CO_2_. The packaging of lentiviruses and their infection were carried out following our published procedure [49]. Cells were subjected to 1.0 μg/mL puromycin selection for 72 h post-infection before further studies.

### 4.3. Microarray Analysis

We treated MDA-MB-231 carrying DOX-inducible SOX7 [17] with DOX for 6, 12 or 24 h in triplicate and isolated total RNA. As a control, we used parental MDA-MB-231 cells treated with DOX for 24 h. RNA was analyzed using the Affymetrix Human Genome U219 Array strips. Gene expression at each time point was normalized to the time point in the control. The microarray data were analyzed by the Kyoto Encyclopedia of Genes and Genomes (i.e., KEGG database) in combination with the Ingenuity Systems software.

### 4.4. Chromatin Immunoprecipitation (ChIP) Assay

Chromatin immunoprecipitation (ChIP) assays were performed as previously reported [50]. Samples immunoprecipitated by a normal IgG (Cat# 7074, Cell Signaling Inc., Beverly, MA, USA) and a SOX7 antibody (Cat# AF2766, R&D Systems) were analyzed with quantitative PCR using the FastStart Universal SYBR Green Master Mix (Roche Diagnostics GmbH, Mannheim, Germany) and the primer sets designed based on the promoter sequences of *SPRY1*, *SLIT2*, *TRIB3* and *MTHFD2* genes. The information of these primer sets is available in Table S1.

### 4.5. Proliferation Assays

We used the Cell Proliferation Reagent WST-1 (Sigma-Aldrich, St. Louis, MO, USA) ‎to evaluate cell proliferation following the procedure provided by the manufacturer. In this assay, cells were infected by lentivirus expressing either shRNAs or cDNAs, with or without the addition of doxycycline (DOX) that could induce SOX7 expression.

### 4.6. Reverse Transcription and Quantitative PCR Analyses

Total cellular RNA was extracted using the TRIzol protocol (Thermo Fisher Scientific Inc., Shanghai, China) and subsequently analyzed by reverse transcription followed by quantitative PCR (RT-PCR). In the reaction of reverse transcription, we added 1 μg of RNA and 0.5 μg/μL of oligo(dT) primer, followed by incubation at 65 °C for 5 min and 4 °C for 2 min. The tubes were then immediately incubated at 42 °C for 30 min and 4 °C. The quantitative PCR analyses were performed using the LightCycler 480 SYBR Green PCR Master Mix and the primers listed in Table S1 in the Roche Lightcycler 480 instrument. The data for each gene were normalized against GAPDH levels.

### 4.7. Reporter Assay

HeLa cells in 12-well plates were cotransfected by 50 ng of reporter constructs, 100 ng of the SOX7 expression vector or an empty vector, and 25 ng of *pCMV-SEAP* (secreted alkaline phosphatase) as a control. Aliquots of the medium from the transfected wells were collected 48 h posttransfection to measure Gluc activity. Fifty microliters of the medium (diluted if necessary) was mixed with 100 μL of substrate solution containing 0.5 μg/mL of coelenterazine (CTZ), 200 mM NaCl, 50 mM Tris·HCl and 0.01% Triton X-100, at pH 8.7. The light emission was measured at a wavelength of 480 nm and normalized against the SEAP expression.

### 4.8. Statistical Analysis 

Data in reporter assays and RT-PCR assays are presented as means ± S.D. Comparisons between two groups on a single parameter were conducted using Student’s *t*-test. Statistical analyses were performed using SigmaStat (Systat Software Inc., Version 12.5, San Jose, CA, USA). The criterion for statistical significance was indicated by asterisks (* as *p* < 0.05, ** as *p* < 0.01, and *** as *p* < 0.001) in the figures.

## Supplementary Materials

Supplementary materials can be found at http://www.mdpi.com/1422-0067/19/5/1451/s1.

## Abbreviations

SOX	SRY-related high mobility group (HMG) box
SPRY1	sprouty homolog 1
SLIT2	slit guidance ligand 2
TRIB3	Tribbles homolog 3
MTHFD2	methylenetetrahydrofolate dehydrogenase (NADP+ dependent)
shRNA	short hairpin RNA
DOX	Doxycycline
GAPDH	glyceraldehyde-3-phosphate dehydrogenase
Gluc	Gaussia luciferase
RT-PCR	reverse-transcription polymerase chain reaction
SEAP	secreted alkaline phosphatase
ChIP	Chromatin Immunoprecipitation
