# Supplementary material for: SOX7 Target Genes and Their Contribution to Its Tumor Suppressive Function
Source: Int J Mol Sci. 2018 May 14;19(5):1451. doi: 10.3390/ijms19051451 (PMC5983648; doi:10.3390/ijms19051451)
Supplement: Supplementary file 1 [file ijms-19-01451-s001.zip › Supplementary Figure 2.pdf]

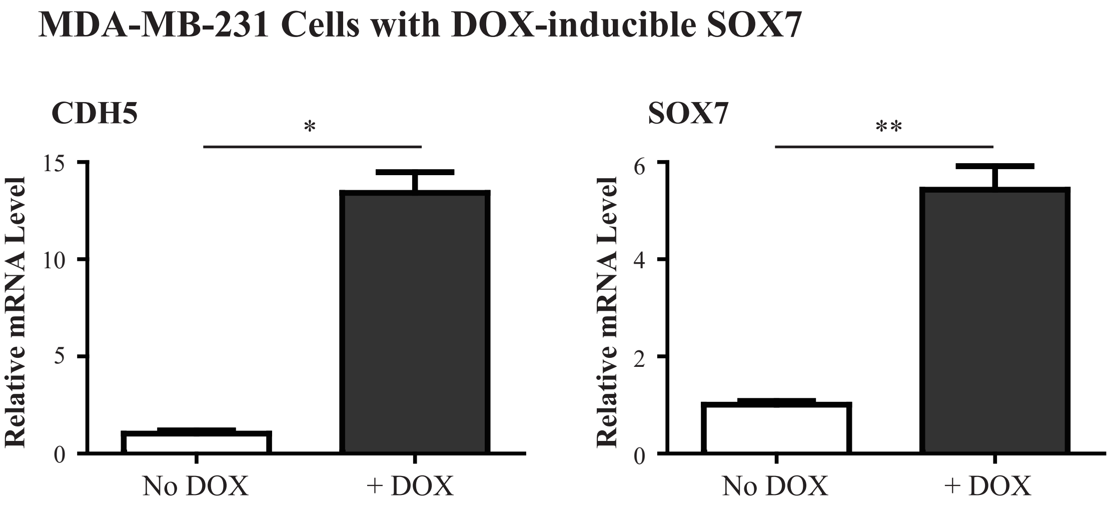

Supplementary Figure 2. Validation of SOX7-mediated transcription of endogenous target genes. Quantitative PCR analyses for CDH5 and SOX7 were carried out, respectively, in reversely transcribed RNA samples extracted from MDA-MB-231 cells with DOX-induced SOX7 expression and its control (No DOX). The data were plotted with the mRNA levels of No DOX samples normalized as 1.
