# Supplementary material for: SOX7 Target Genes and Their Contribution to Its Tumor Suppressive Function
Source: Int J Mol Sci. 2018 May 14;19(5):1451. doi: 10.3390/ijms19051451 (PMC5983648; doi:10.3390/ijms19051451)
Supplement: Supplementary file 1 [file ijms-19-01451-s001.zip › Supplementary Figure 3.pdf]

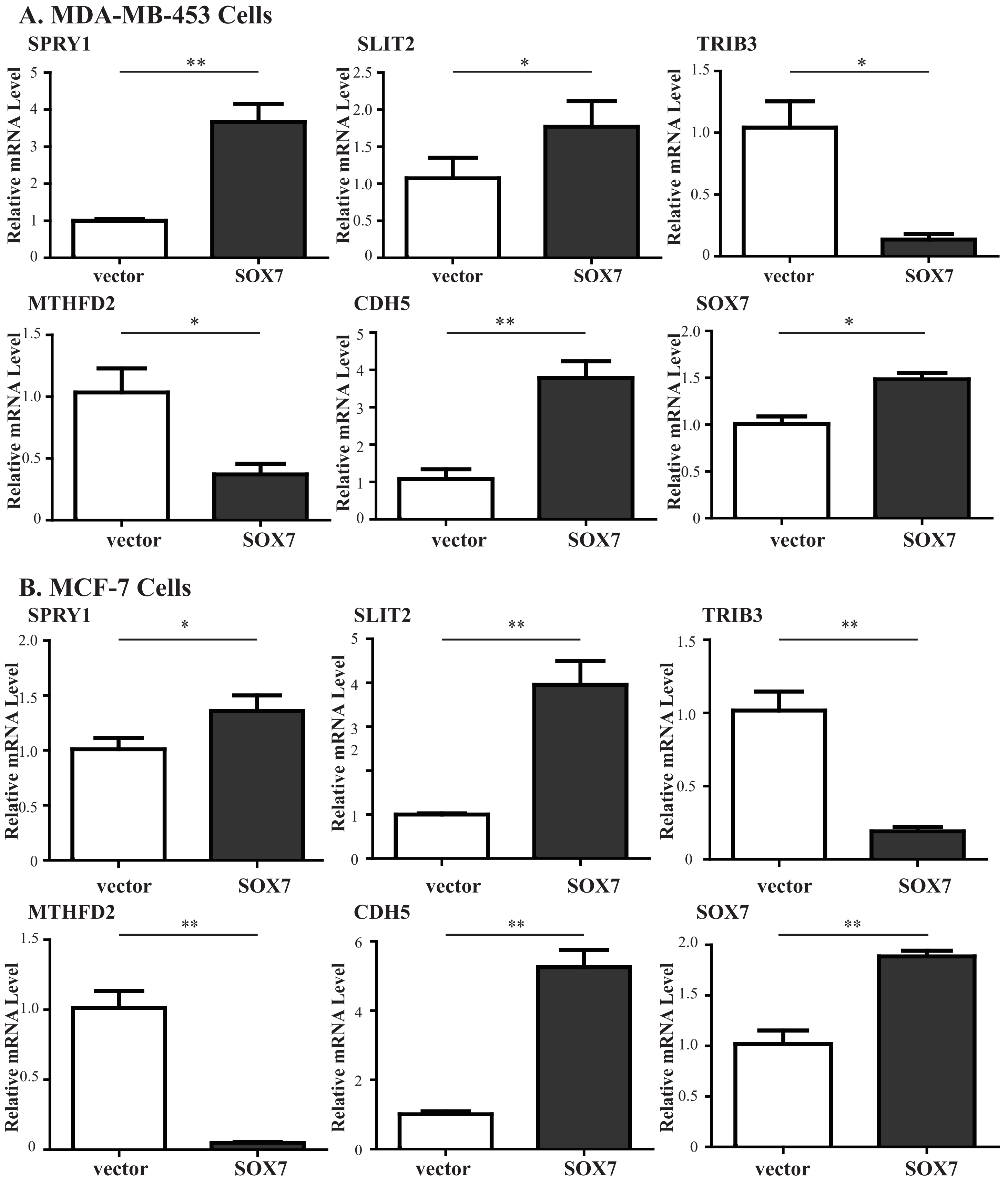

Supplementary Figure 3. Evaluation of potential SOX7 target genes in MDA-MB-453 and MCF-7 cell lines. Lentivirus carrying SOX7 expression cassette or an empty vector (as a control) were used to infect (A) MDA-MB-453 and (B) MCF-7 cells. After 72 h, cells were collected followed by total RNA extraction and reverse transcription using poly(dT). Then, quantitative PCR analyses on the reversely transcribed samples were carried out using primer pairs for SPRY1, SLIT2, TRIB3, MTHFD2, CDH5 and SOX7, respectively. The data were plotted with the mRNA levels of vector control samples normalized as 1. * as $p < 0.05$, ** as $p < 0.01$.
