# Supplementary material for: SOX7 Target Genes and Their Contribution to Its Tumor Suppressive Function
Source: Int J Mol Sci. 2018 May 14;19(5):1451. doi: 10.3390/ijms19051451 (PMC5983648; doi:10.3390/ijms19051451)
Supplement: Supplementary file 1 [file ijms-19-01451-s001.zip › Supplementary Table 1.docx]

**Supplementary Table 1.** Oligonucleotides used in (1) amplifying the promoters of SPRY1, SLIT2, TRIB3 and MTHFD2, (2) amplifying the cDNAs of TRIB3 and MTHFD2, (3) conducting quantitative PCR, and (4) generating shRNA constructs (including target site sequences). The underlined sequences correspond to the sites of restriction enzymes.

| 1. **Primers used to amplify and subclone the 2kb promoters of four SOX7 target genes** | | |
| --- | --- | --- |
| **Name** | **Sequence (5’ to 3’)** | **Purpose** |
| hSPRY1prmt-for-1 | CAG GGC TCA CTT TCC ATT CCA C | Human SPRY1 promoter nest PCR,  the first step, forward primer |
| hSPRY1prmt-rev-1 | GTG CTG ATA ATC ACT CTG CAA AG | Human SPRY1 promoter nest PCR,  the first step, reverse primer |
| hSPRY1prmt-for-2-BamHI | cgag GGA TCC ACA ATG TAA TTA AAC TTA CCA AGT G | Human SPRY1 promoter nest PCR,  the second step, forward primer |
| hSPRY1prmt-rev-2-HindIII | ggac aag ctt CTA CGC AGA ATT TCA GCT CCT TG | Human SPRY1 promoter nest PCR,  the second step, reverse primer |
| hSLIT2prmt-for-1 | AGC CGC GTT CAA AGC CGA CCT CT | Human SLIT2 promoter nest PCR,  the first step, forward primer |
| hSLIT2prmt-rev-1 | TCT TTC CCC GCC GCC TCC TTC CTC | Human SLIT2 promoter nest PCR,  the first step, reverse primer |
| hSLIT2prmt-for-2-XbaI | ccag tctaga GAC TAT CTC CGA GCC CAG GTC AC | Human SLIT2 promoter nest PCR,  the second step, forward primer |
| hSLIT2prmt-rev-2-HindIII | ggac aagctt CCT CTC CAC CCT CCT CTG GC | Human SLIT2 promoter nest PCR,  the second step, reverse primer |
| hTRIB3prmt-for-1 | GAA GGC TCT GGG ACA CAG GTG | Human TRIB3 promoter nest PCR,  the first step, forward primer |
| hTRIB3prmt-rev-1 | CAG ACA TCA GCC ACG GAC TCT C | Human TRIB3 promoter nest PCR,  the first step, reverse primer |
| hTRIB3prmt-for-2-BamHI | cgag GGA TCC TGC Ctc cca tcc agc ctc tgc c | Human TRIB3 promoter nest PCR,  the second step, forward primer |
| hTRIB3prmt-rev-2-SacI | c acg gag ctc CGG ACT CTC CCT ATC ACC TTG G | Human TRIB3 promoter nest PCR,  the second step, reverse primer |
| hMTHFD2prmt-for-1 | AGC CCC CAG AAC TGT TAG AAA ATA | Human MTHFD2 promoter nest PCR,  the first step, forward primer |
| hMTHFD2prmt-rev-1 | CAG CCG GGC AGC CAA AGC AGA C | Human MTHFD2 promoter nest PCR,  the first step, reverse primer |
| hMTHFD2prmt-for-2-XbaI | ccag tct aga GGC AGC CCT AGC CAA CTT ATA C | Human MTHFD2 promoter nest PCR,  the second step, forward primer |
| hMTHFD2prmt-rev-2-SacI | cacg gagctc CCA CGC GGT TAT ACT GCG GC | Human MTHFD2 promoter nest PCR,  the second step, reverse primer |

| 1. **Primers used to amplify and subclone TRIB3 and MTHFD2 cDNAs** | | |
| --- | --- | --- |
| **Name** | **Sequence** | **Purpose** |
| hTRIB3-for | CGG GCC CAC GCG GAA CGA C | Human TRIB3 cDNA nest PCR,  the first step, forward primer |
| hTRIB3-rev | GGG CTT GGC ACC TGA TAA G | Human TRIB3 cDNA nest PCR,  the first step, reverse primer |
| hTRIB3-BamHI-4U | cgag GGA TCC CGA GCC ACC CCT CTG GCT GCT C | Human TRIB3 cDNA nest PCR,  the second step, forward primer |
| hTRIB3-EcoRI-1077L | cacg gaa ttc CTA GCC ATA CAG AAC CAC TTC TC | Human TRIB3 cDNA nest PCR,  the second step, reverse primer |
| hMTHFD2-for | CTT CCC TCC CGG CGC AGT CAC C | Human MTHFD2 cDNA nest PCR,  the first step, forward primer |
| hMTHFD2-rev | CTA GAA AAG GCG AAT GTG TAA C | Human MTHFD2 cDNA nest PCR,  the first step, reverse primer |
| hMTHFD2-BamHI-4U | cgag GGA TCC GCT GCG ACT TCT CTA ATG TCT G | Human MTHFD2 cDNA nest PCR,  the second step, forward primer |
| hMTHFD2-EcoRI-1053L | cacg gaa ttc TTA ATT AGT GGC TAC CCC AAG C | Human MTHFD2 cDNA nest PCR,  the second step, reverse primer |

| 1. **Primers used in quantitative PCR** | | |
| --- | --- | --- |
| **Name** | **Sequence** | **Purpose** |
| hSPRY1-21U | TGG CAG TGG CAG TTC GTT AGT TGT | Quantitative PCR for SPRY1,  forward primer |
| hSPRY1-201L | TGT CCG AGG AGC AGG TCT TTT CA | Quantitative PCR for SPRY1,  reverse primer |
| hSLIT2-1936U | GCA CCA GGG GCA TTT GAT ACT C | Quantitative PCR for SLIT2,  forward primer |
| hSLIT2-2063L | TTT CCC GTG ACA ATT CTC TTC TTT | Quantitative PCR for SLIT2,  reverse primer |
| hTRIB3-765U | GGC CGG CCA CTA CCC CTT CC | Quantitative PCR for TRIB3,  forward primer |
| hTRIB3-1033L | CTT CGT CCA GCC CCA GTC CAT C | Quantitative PCR for TRIB3,  reverse primer |
| hMTHFD2-863U | AGT TGG TTG GAG ATG TGG ATT TTG | Quantitative PCR for MTHFD2,  forward primer |
| hMTHFD2-1452L | CTA GAA AAG GCG AAT GTG TAA C | Quantitative PCR for MTHFD2,  reverse primer |
| huSOX7-149U | ACG CCT TCA TGG TTT GGG | Quantitative PCR for SOX7,  forward primer |
| huSOX7-370L | GCT TGG CCT GCT TCT TCC | Quantitative PCR for SOX7,  reverse primer |
| GAPDH-for | GGG AGC CAA AAG GGT CAT | Quantitative PCR for GAPDH,  forward primer |
| GAPDH-rev | GAG TCC TTC CAC GAT ACC AA | Quantitative PCR for GAPDH,  reverse primer |
| huCDH5-944U | TCA CCC AGA CCA AGT ACA CAT T | Quantitative PCR for CDH5,  forward primer |
| huCDH5-1054L | ACT TGG TCA TCC GGT TCT GG | Quantitative PCR for CDH5,  reverse primer |

| 1. **Oligonucleotides used to generate shRNA constructs targeting SPRY1 and SLIT2** | | |
| --- | --- | --- |
| **Name** | **Sequence** | **Purpose** |
| SPRY1-227-H3L | CAGC AAGCTT GAA TAT TGG TAT GAT TTC ATG AGT CC GGG ATC TCT ATC ACT GAT AGG GAA C | Primers for generating shSPRY1-1;  target site: GGA CTC ATG AAA TCA TAC CAA TT |
| SPRY1-227-2a | AGC TT AAT TGG TAT GAT TTC ATG AGT CC T TTT TG |  |
| SPRY1-227-2b | AAT TC A AAA A GGA CTC ATG AAA TCA TAC CAA TT A |  |
| SPRY1-516-H3L | CAGC AAGCTT GAA TAC TTG TGC TGT GTC AGG TCC GGG ATC TCT ATC ACT GAT AGG GAA C | Primers for generating shSPRY1-2;  target site: GGA CCT GAC ACA GCA CAA GTT |
| SPRY1-516-2a | AGC TT AAC TTG TGC TGT GTC AGG TCC T TTT TG |  |
| SPRY1-516-2b | AAT TC A AAA A GGA CCT GAC ACA GCA CAA GTT A |  |
| SLIT2-420-H3L | CAGC AAGCTT GAA TAA GCT TTC CTT GGG ATT GCC GGG ATC TCT ATC ACT GAT AGG GAA C | Primers for generating shSLIT2-1;  target site: GGC AAT CCC AAG GAA AGC TTT |
| SLIT2-420-2a | AGC TT AAA GCT TTC CTT GGG ATT GCC T TTT TG |  |
| SLIT2-420-2b | AAT TC A AAA A GGC AAT CCC AAG GAA AGC TTT A |  |
| SLIT2-2548-H3L | CAGC AAGCTT GAA TAT TGC TAG ATG TGA TAA TGC GGG ATC TCT ATC ACT GAT AGG GAA C | Primers for generating shSLIT2-2;  target site: GCA TTA TCA CAT CTA GCA ATT |
| SLIT2-2548-2a | AGC TT AAT TGC TAG ATG TGA TAA TGC T TTT TG |  |
| SLIT2-2548-2b | AAT TC A AAA A GCA TTA TCA CAT CTA GCA ATT A |  |
| shCont-H3L | CA GCA AGC TTG AA TAT GAC GTA ATA GAG TAG TCCC A AAC AAG GCT TTT CTC CAA GGG ATA | Primers for generating shCont;  A scrambled “target” site: GGGA CTA CTC TAT TAC GTC ATT |
| shCont-2a | AGC TT AAT GAC GTA ATA GAG TAG TCCC T TTT TG |  |
| shCont-2b | AAT TC A AAA A GGGA CTA CTC TAT TAC GTC ATT A |  |

| 1. **Primers for ChIP assays** | | |
| --- | --- | --- |
| **Name** | **Sequence** | **Purpose** |
| SPRY1-PCR-U | TGCTCTGTGTTCTTCAGAGGC | PCR primers on the SPRY1 promoter; predicted PCR product size: 132 bps. |
| SPRY1-PCR-L | AGCAAGTGGTTCTGTTGAAACT |  |
| SLIT2-PCR-U | CTTCCGCGCCTTCTAGCTTC | PCR primers on the SLIT2 promoter; predicted PCR product size: 106 bps. |
| SLIT2-PCR-L | TCTGCAAATCCGATGCCAGT |  |
| TRIB3-PCR-U | ACCCCAAATCAAGTAGCACCA | PCR primers on the TRIB3 promoter; predicted PCR product size: 104 bps. |
| TRIB3-PCR-L | CGGATGCTCAGTGGAGTGTAAA |  |
| MTHFD2-PCR-U | TGGACAGTTTTAACATTCTAGGTCT | PCR primers on the MTHFD2 promoter; predicted PCR product size: 101 bps. |
| MTHFD2-PCR-L | AGGTGATTTTCCTAAACCAATGAGT |  |
