# Supplementary material for: SOX7 Target Genes and Their Contribution to Its Tumor Suppressive Function
Source: Int J Mol Sci. 2018 May 14;19(5):1451. doi: 10.3390/ijms19051451 (PMC5983648; doi:10.3390/ijms19051451)
Supplement: Supplementary file 1 [file ijms-19-01451-s001.zip › Supplementary Table 3.docx]

**Supplementary Table 3. Predicted SOX7 binding sites in the positive or negative strands of the promoters of the SPRY1, SLIT2, TRIB3 and MTHFD2 genes.** The core consensus binding sites as 5’-(A/T)(A/T)CAA(A/T)G-3’ (Wegner, M. 1999. ***Nucleic Acids Res*** 27, 1409) are underlined. The positions of these core sites are provided with the first nucleotide of a transcription start site (TSS) designated as “+1”, and the first nucleotide downstream TSS as “-1”.

| **Genes** | **#** | **SOX7 Binding Sites**  **(5’ - 3’)** | **Strands** | **Positions of**  **the Core sites** |
| --- | --- | --- | --- | --- |
| **SPRY1** | 1 | TGAAA**AACAATG**TATAC | Positive | -1985 to -1979 |
|  | 2 | ATTTC**TACAAAG**TCTTA | Positive | -1949 to -1943 |
|  | 3 | TTTCA**TTCAATG**TCTTT | Positive | -1905 to -1899 |
|  | 4 | AAAAA**TACAATG**AAATC | Positive | -1376 to -1370 |
|  | 5 | TTCTT**AACAATG**CTCTG | Positive | -1235 to -1229 |
|  | 6 | ATGTA**CTTTGAA**CTAAA | Negative | -1074 to -1068 |
|  | 7 | CACAA**CTTTGAT**AATAA | Negative | -849 to -843 |
|  | 8 | CTGAA**CTTTGTT**GTAAA | Negative | -458 to -452 |
| **SLIT2** | 1 | CGGAA**TTCAAAG**CCTGG | Positive | -1090 to -1084 |
|  | 2 | GCCCA**CTTTGAT**CGGGG | Negative | -291 to -285 |
| **TRIB3** | 1 | TTTCT**CTTTGTA**AAGAC | Negative | -1466 to -1460 |
|  | 2 | GTCTC**CATTGTT**TACAC | Negative | -864 to -858 |
| **MTHFD2** | 1 | ATACA**TACAAAG**CATTA | Positive | -1989 to -1983 |
|  | 2 | GCTAA**CTTTGAT**CACGT | Negative | -1321 to -1315 |
|  | 3 | CTGTT**CTTTGTT**TGTTT | Negative | -1199 to -1193 |
