# Supplementary material for: SOX7 Target Genes and Their Contribution to Its Tumor Suppressive Function
Source: Int J Mol Sci. 2018 May 14;19(5):1451. doi: 10.3390/ijms19051451 (PMC5983648; doi:10.3390/ijms19051451)

Supplementary Figure 1. Signaling pathways affected by SOX7 induction in MDA-MB-231 cells.

The Affymetrix Human Genome U219 Array strips were used to analyze RNA samples from MDA-MB-231 cells with DOX-induced SOX7 expression. The microarray data were analyzed by the Kyoto Encyclopedia of Genes and Genomes (i.e. KEGG database). The affected pathways are listed below and schematically presented in the following pages. The regulatory genes with POSITIVE fold changes are highlighted in PINK and genes with NEGATIVE fold changes are in GREEN.

List of affected signaling pathways:

1. Apoptosis Pathways
2. p53 Signaling Pathway
3. ERBB Signaling Pathway
4. mTOR Signaling Pathways
5. TGF-β Signaling Pathways
6. Toll-Like Receptor Signaling Pathway
7. JAK-STAT Signaling Pathway
8. Insulin Signaling Pathway
9. MAP Signaling Pathway
10. Chemokine Signaling Pathway
11. Pathways in Cancer
12. Pancreatic Cancer
13. Glioma
14. Bladder Cancer
15. Small Cell Lung Cancer
16. Chronic Myeloid Leukemia
17. Endocytosis
18. Osteoclast Differentiation
19. Focal Adhesion
20. ECM-Receptor Interaction
21. Cell Adhesion Molecules
22. Complement and Coagulation Cascades
23. NOD-Like Receptor Signaling Pathway
24. Hematopoietic Cell Lineage
25. FcγR-Mediated Phagocytosis
26. Type I Diabetes Mellitus
27. Bacterial Invasion of Epithelial Cells
28. Malaria
29. Amoebiasis
30. Measles
31. Influenza A
32. Rheumatoid Arthritis

Apoptosis Pathways

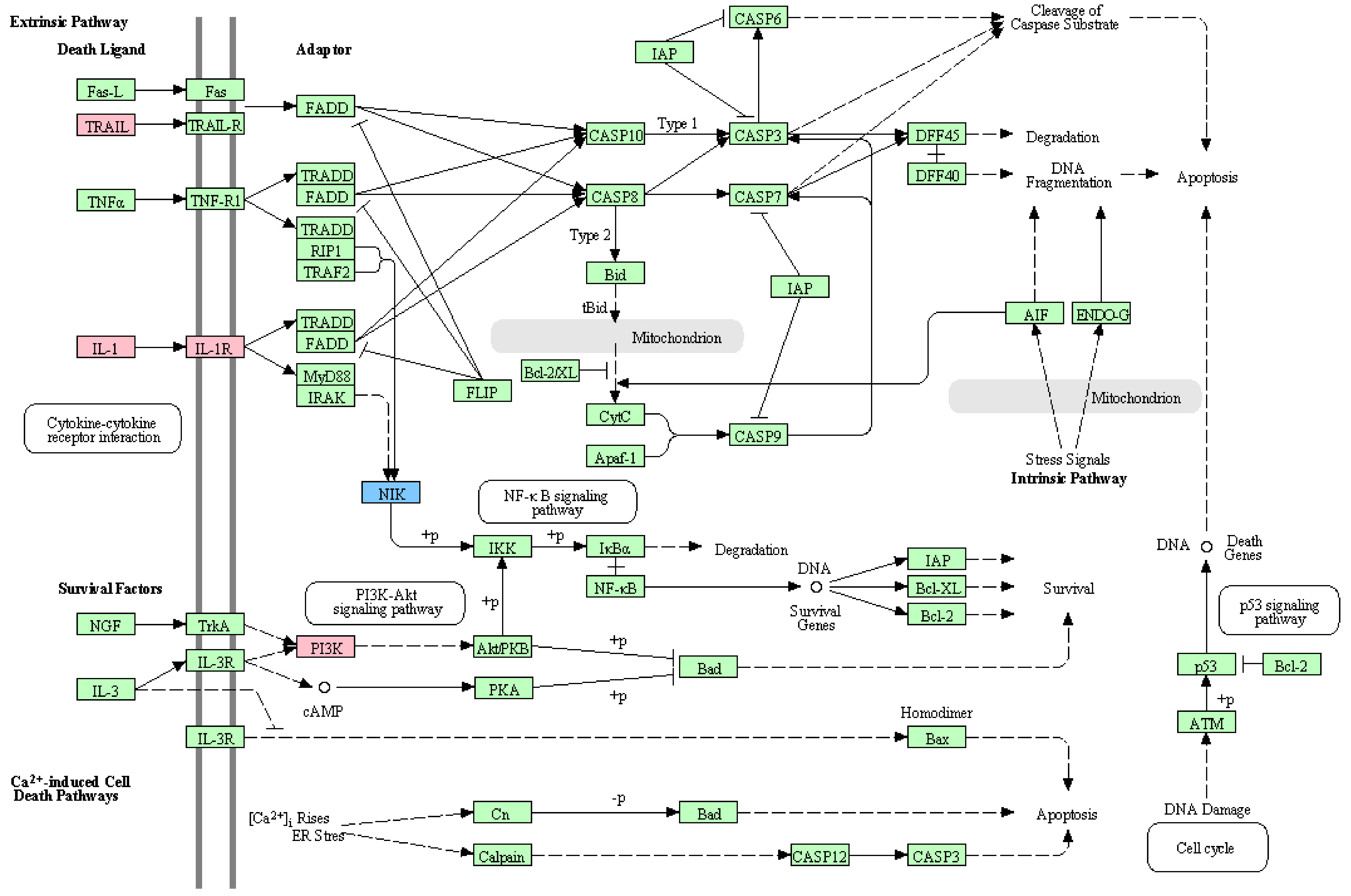

p53 Signaling Pathway

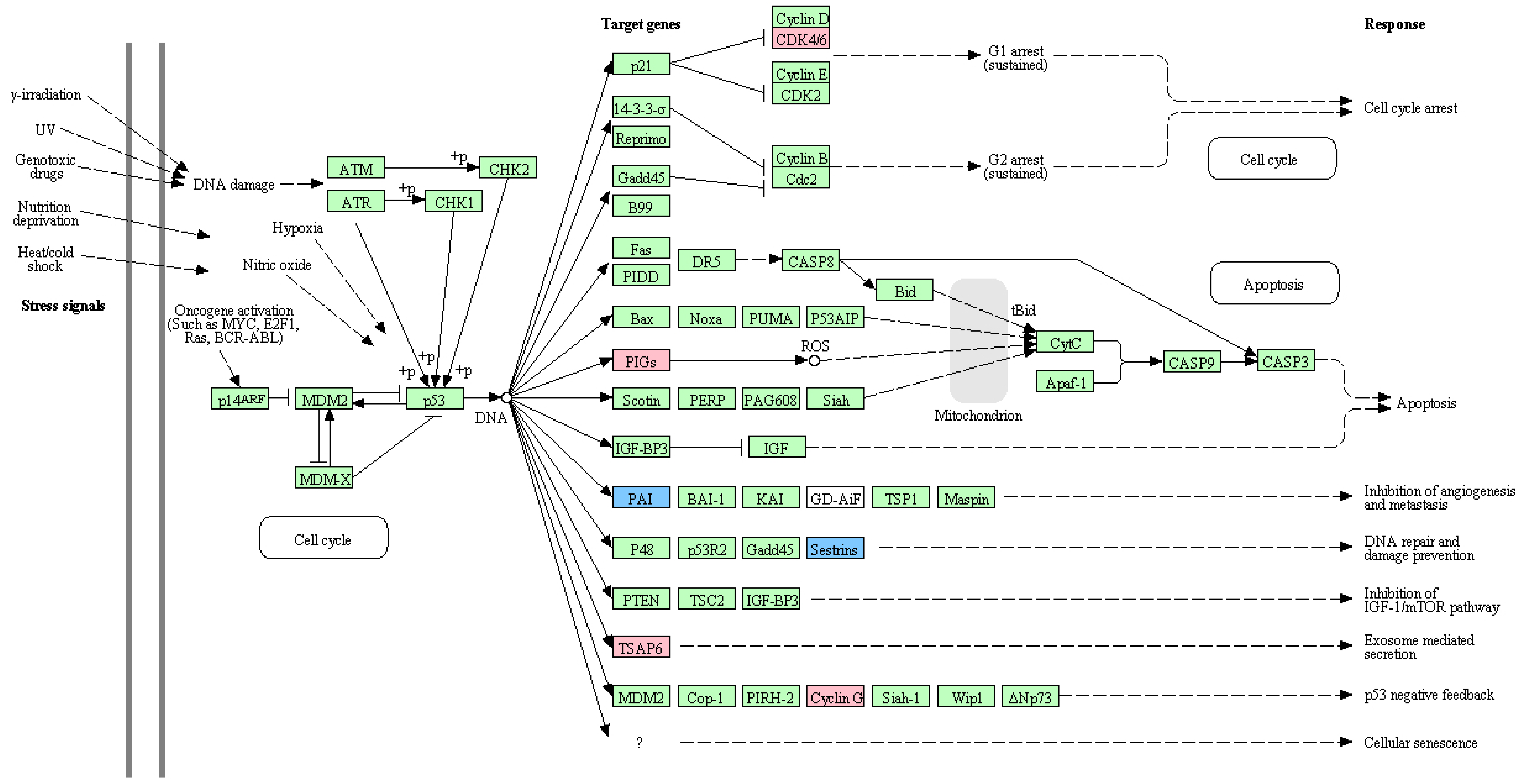

ERBB Signaling Pathway

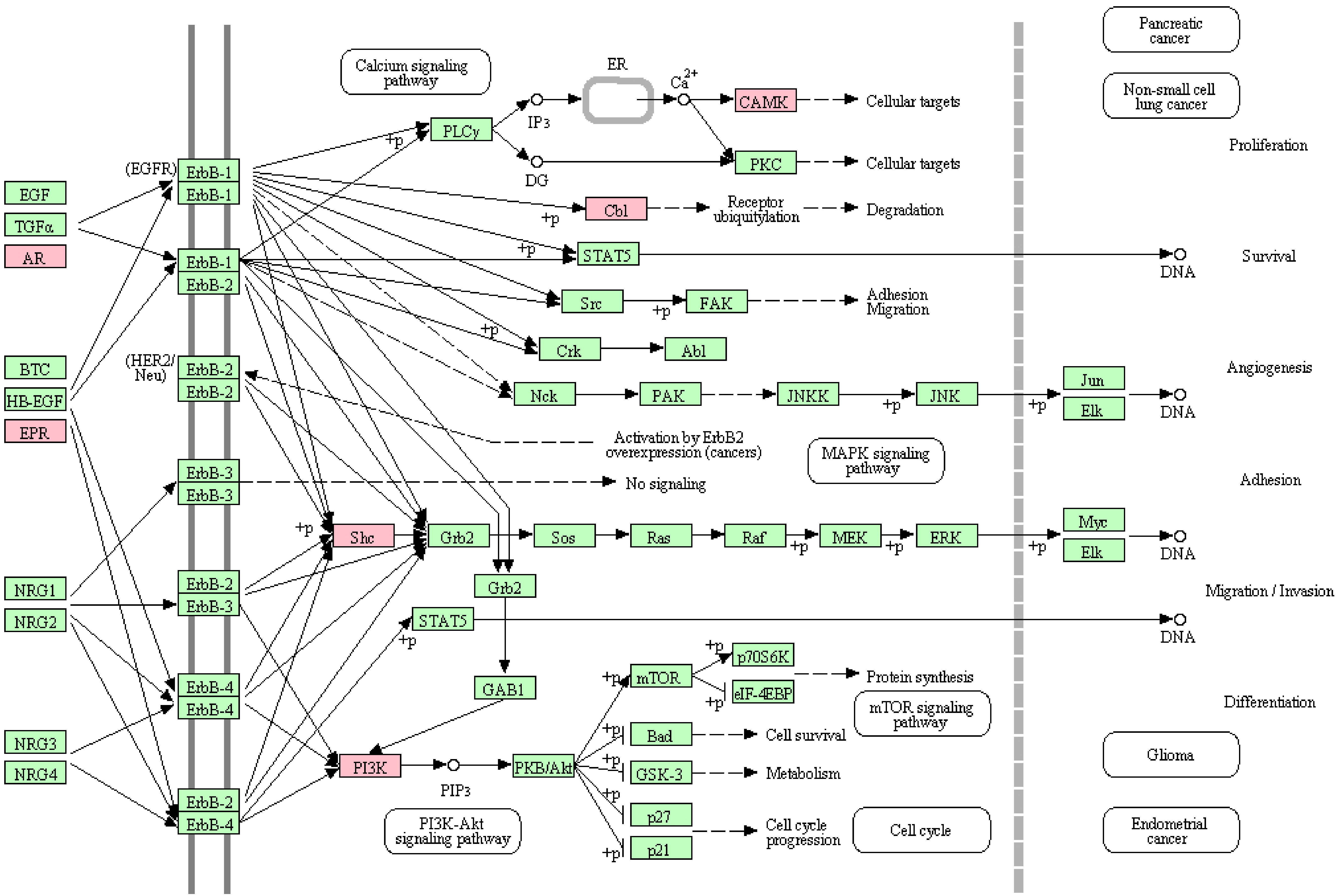

mTOR Signaling Pathways

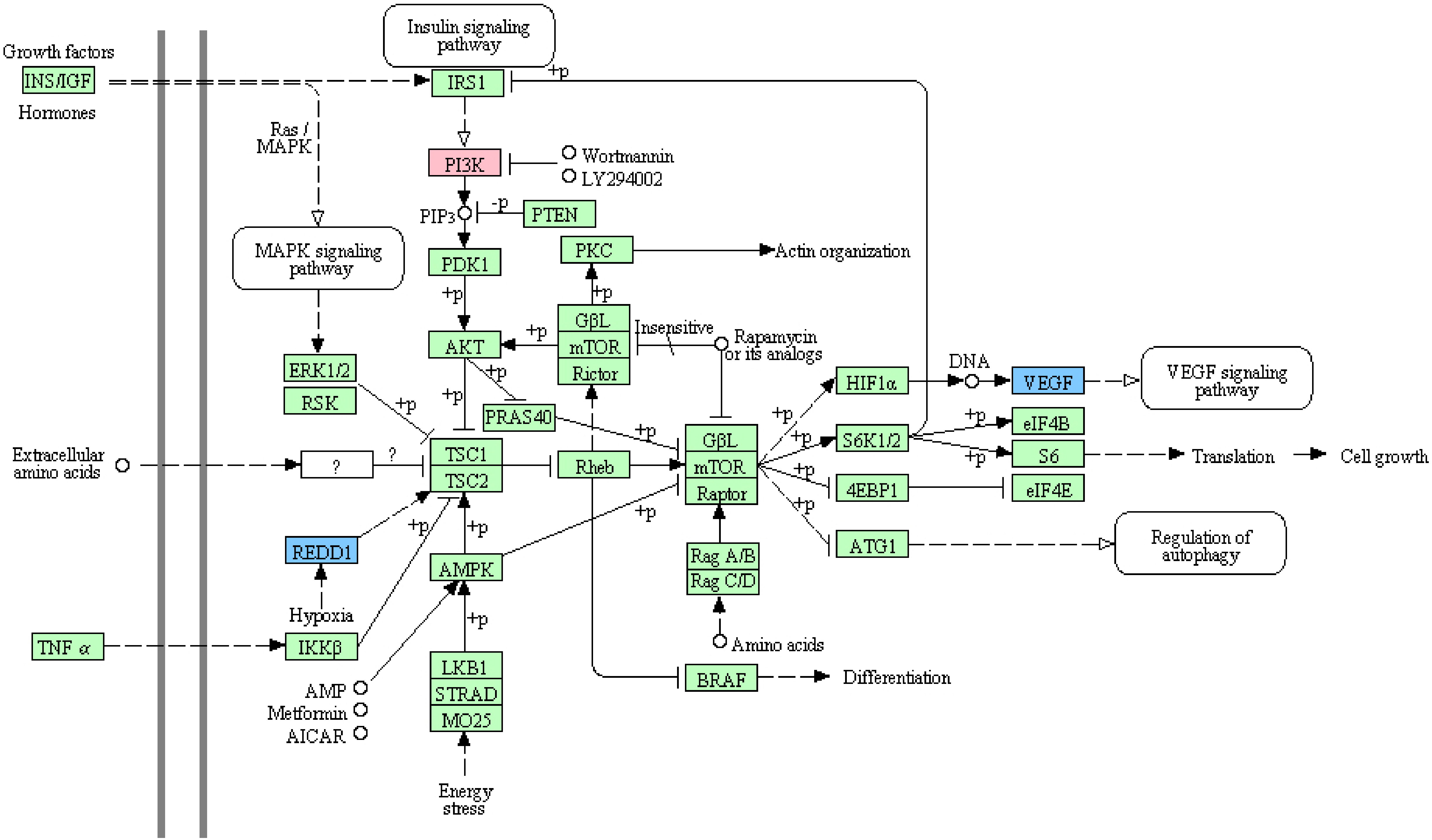

TGF-β Signaling Pathways

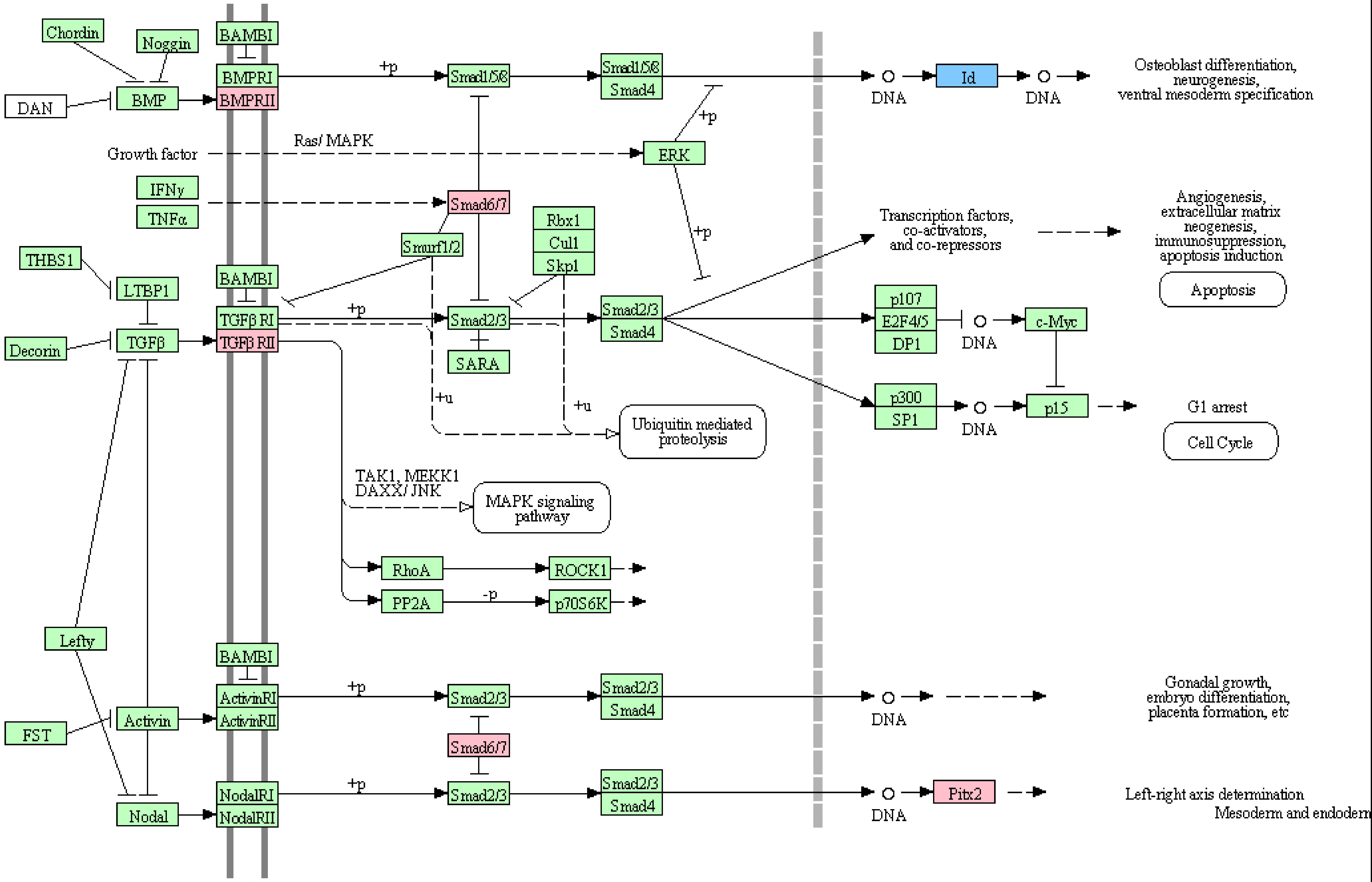

Toll-Like Receptor Signaling Pathway

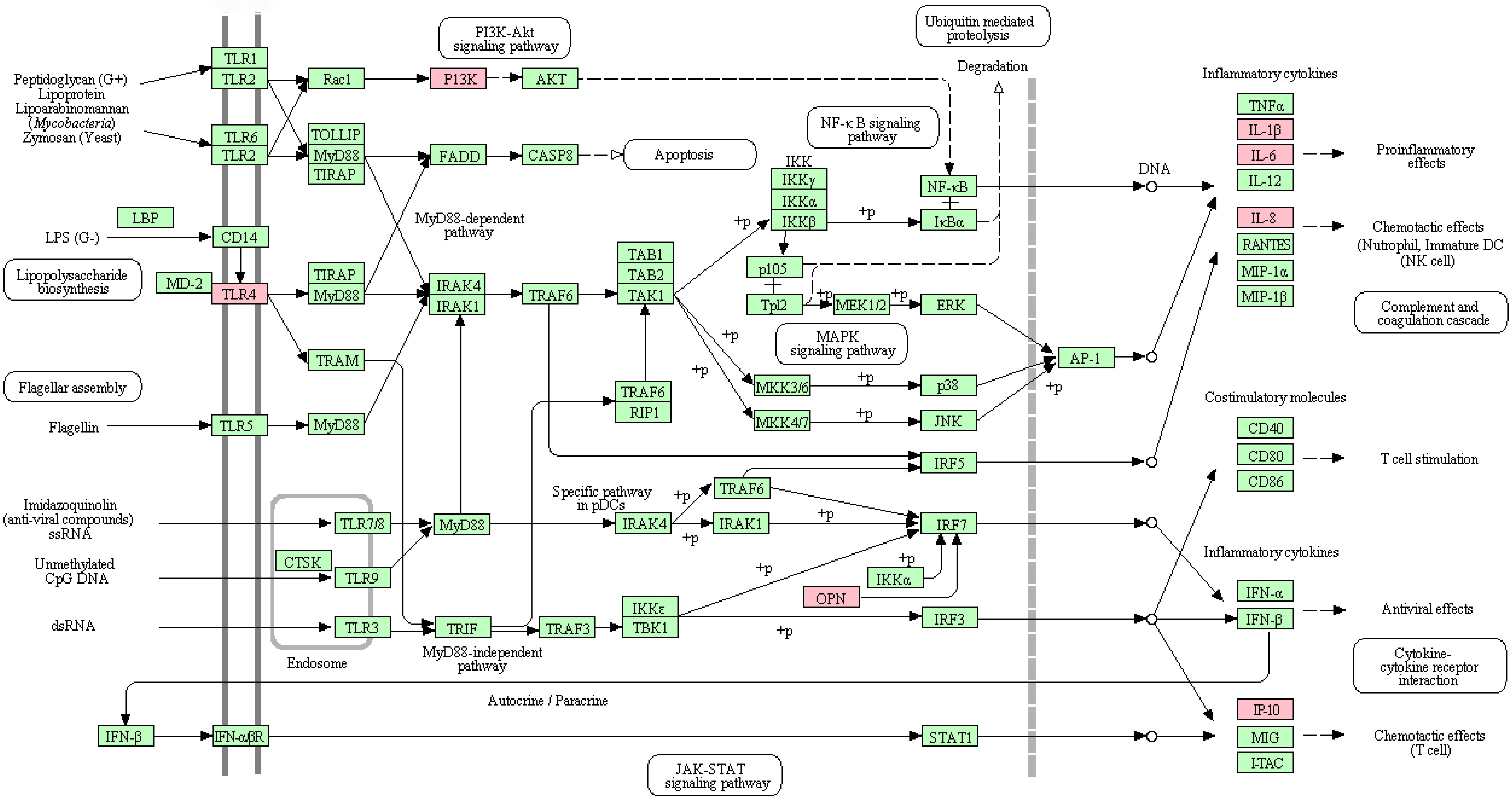

JAK-STAT Signaling Pathway

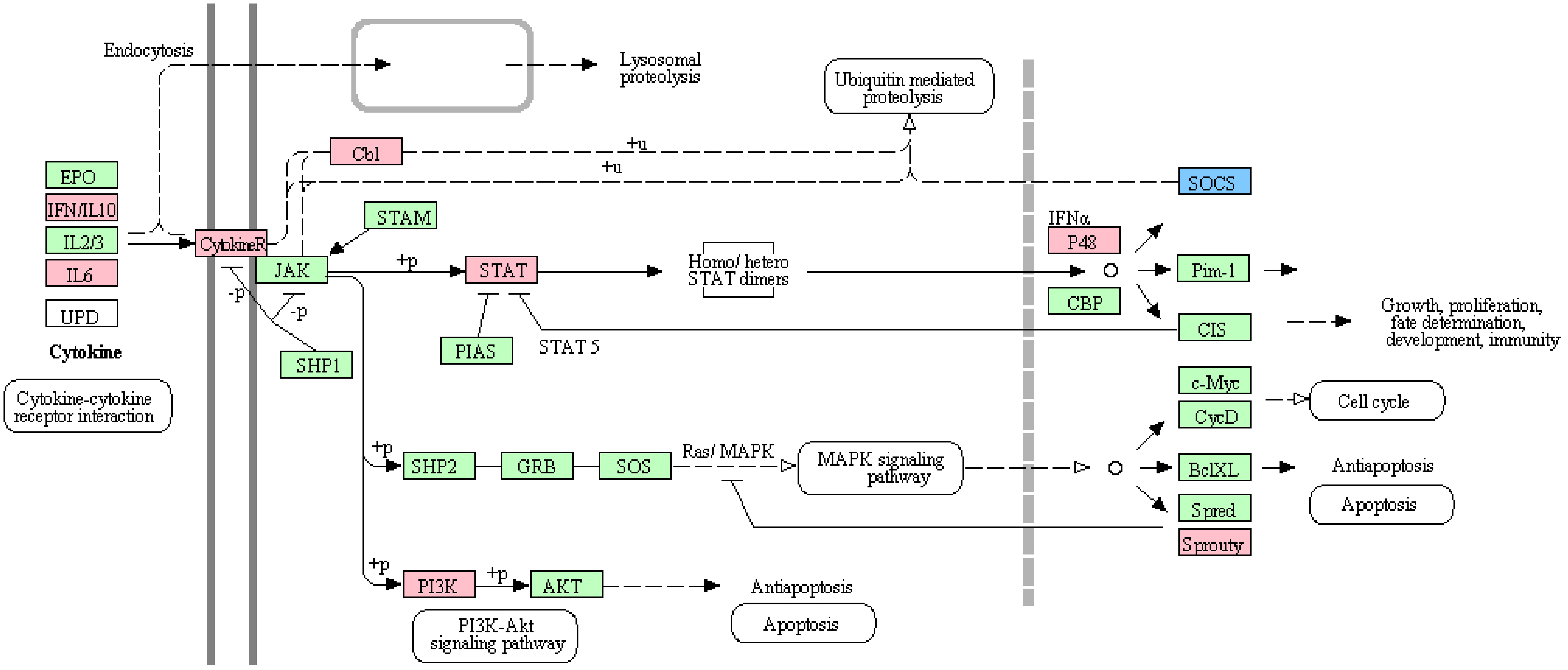

Insulin Signaling Pathway

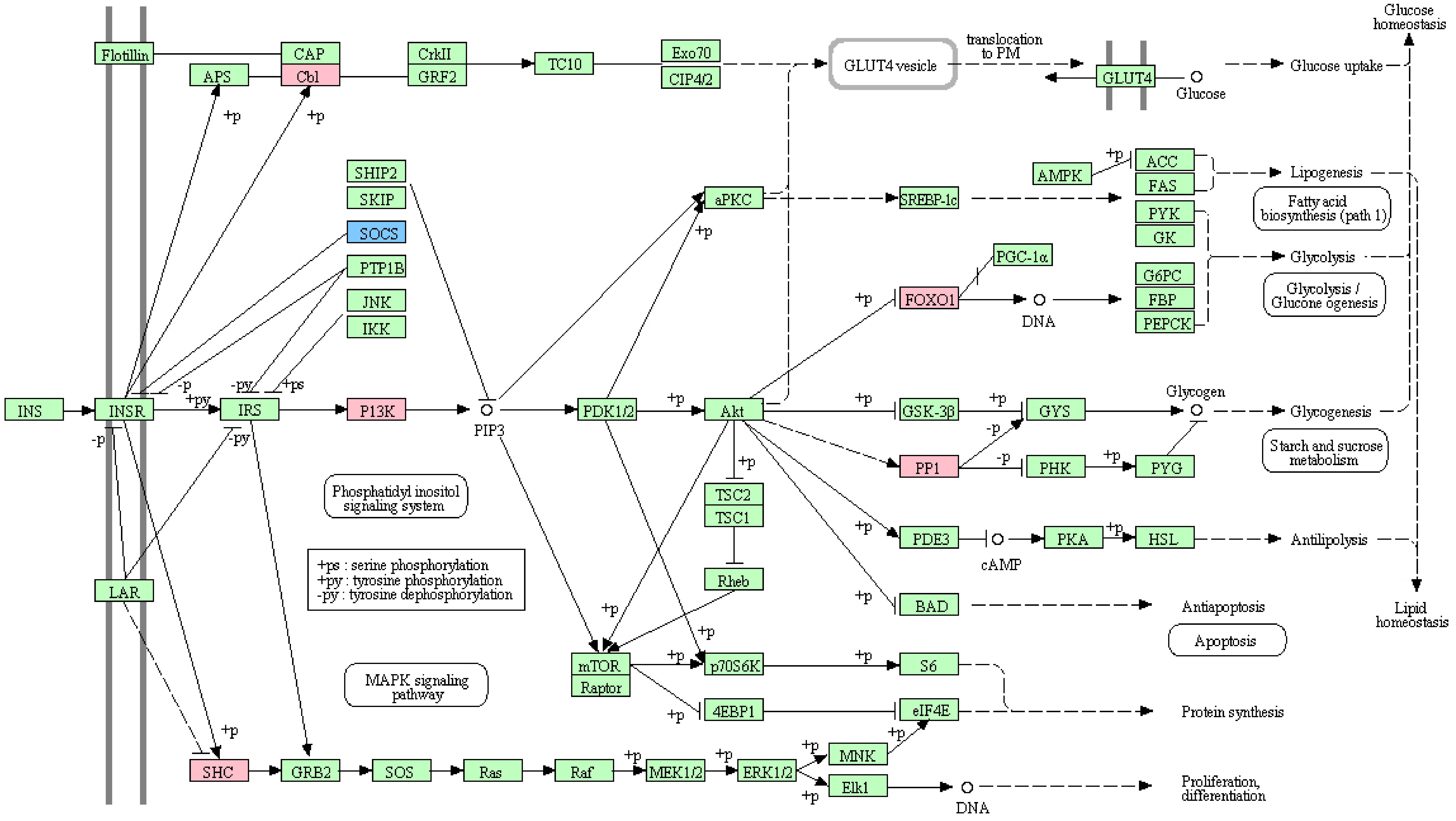

MAP Signaling Pathway

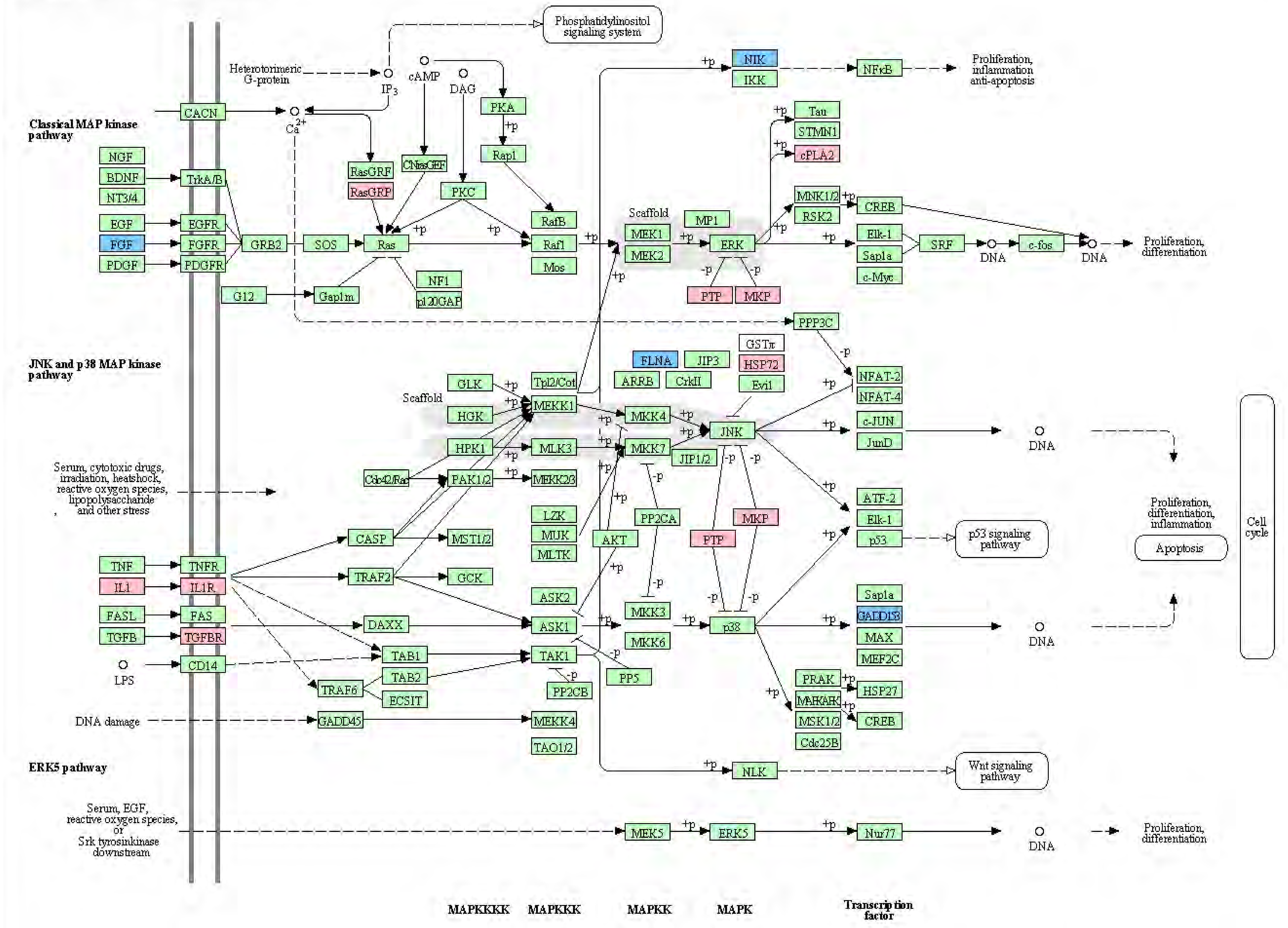

Chemokine Signaling Pathway

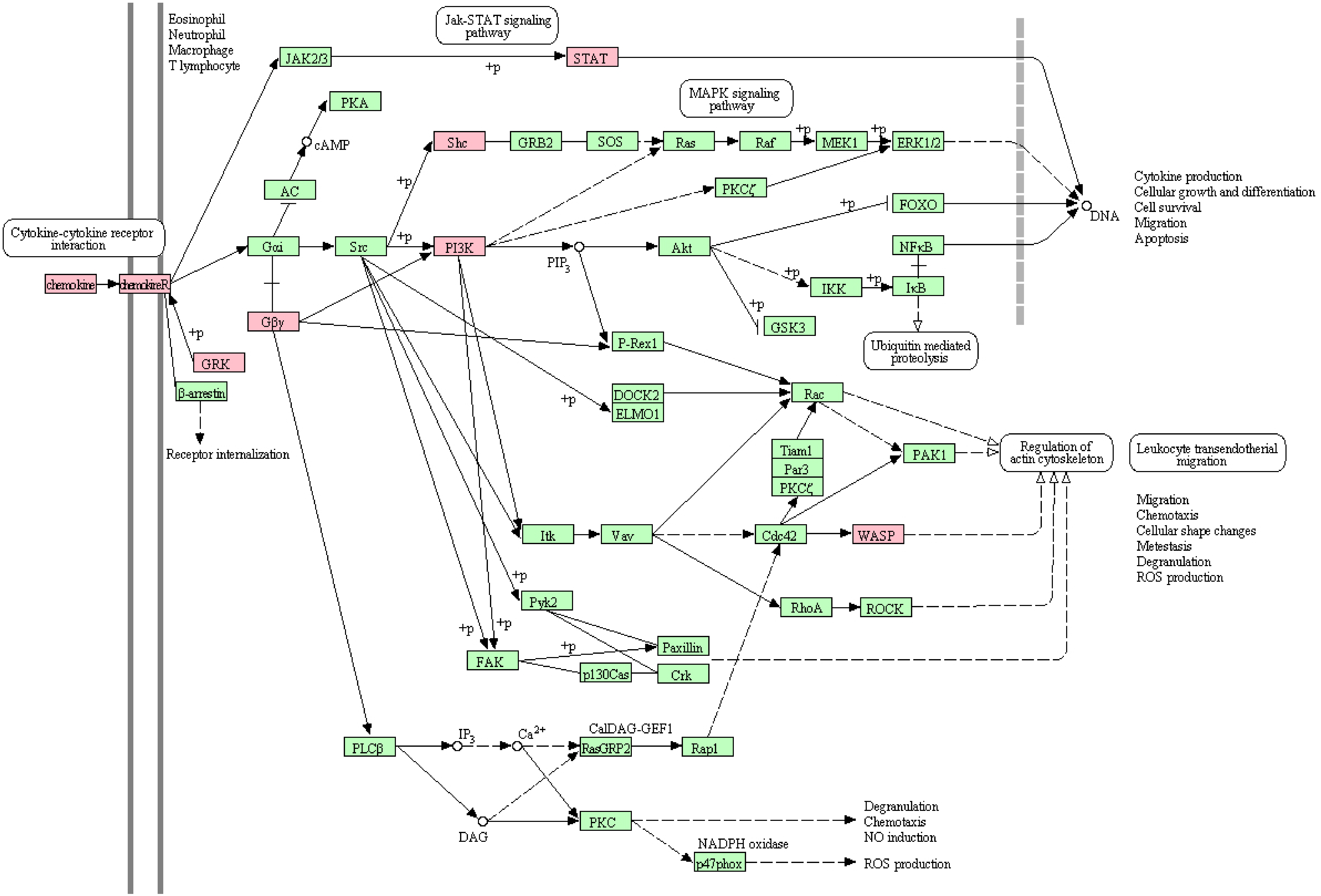

Pathways in Cancer

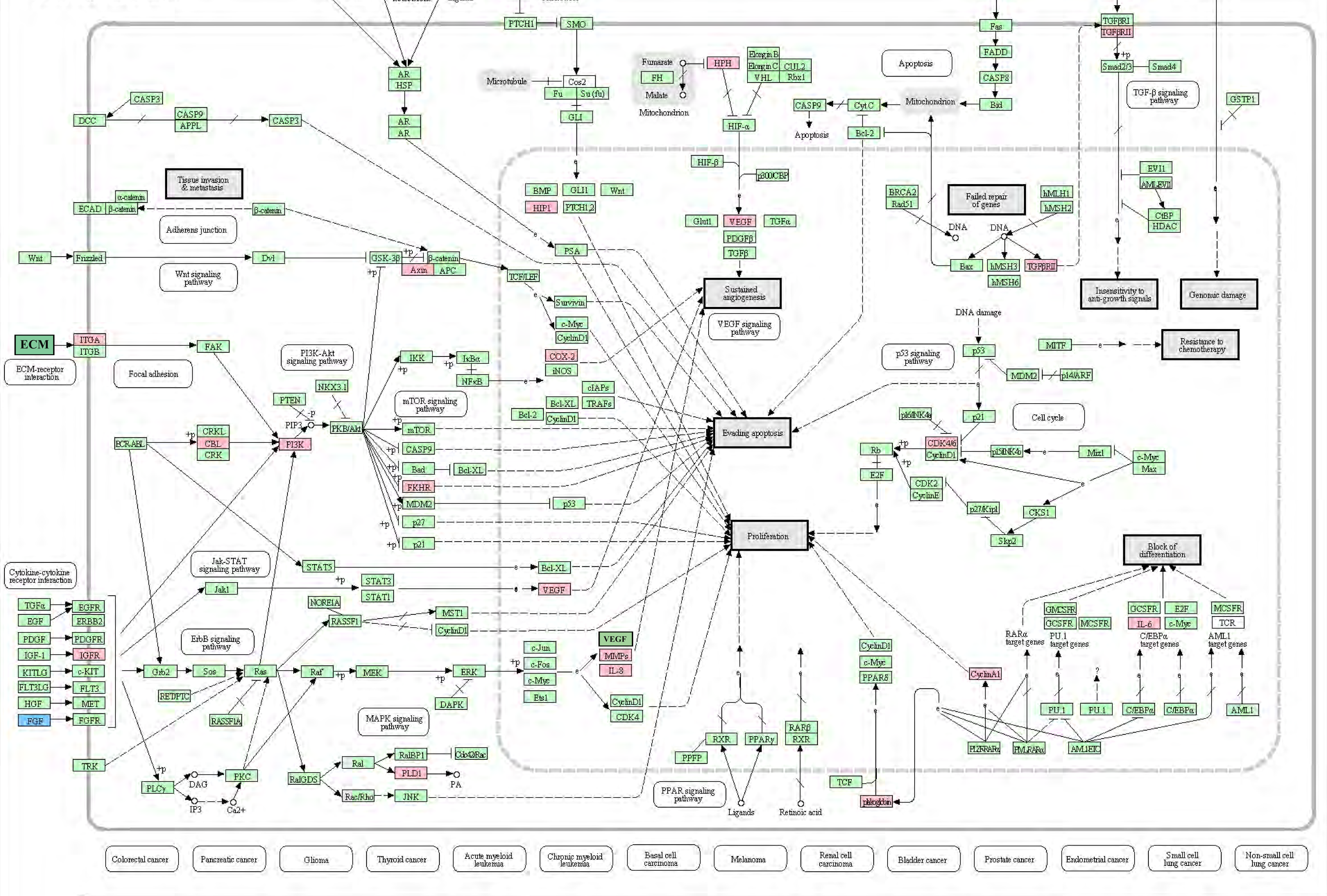

Pancreatic Cancer

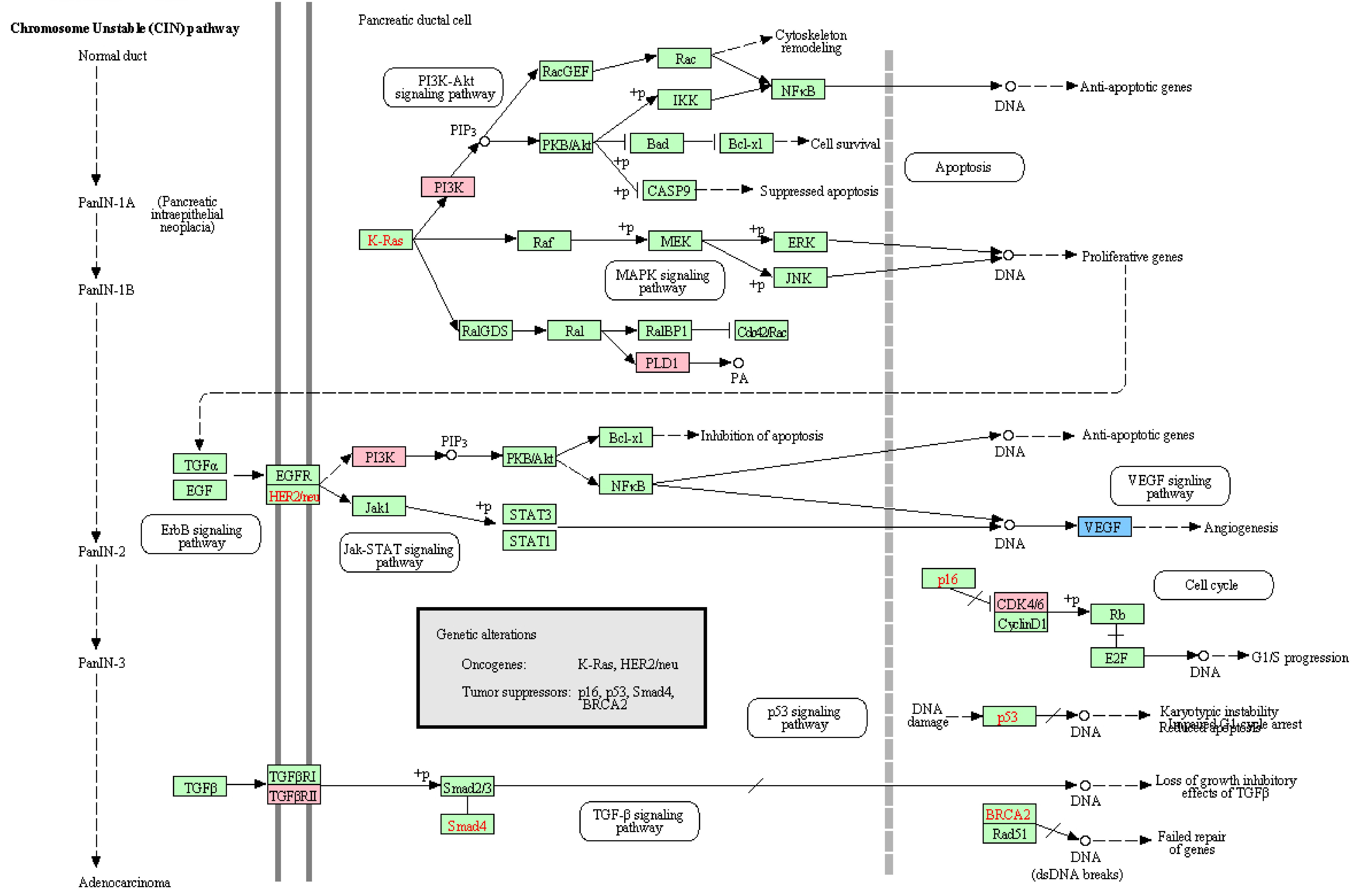

Glioma

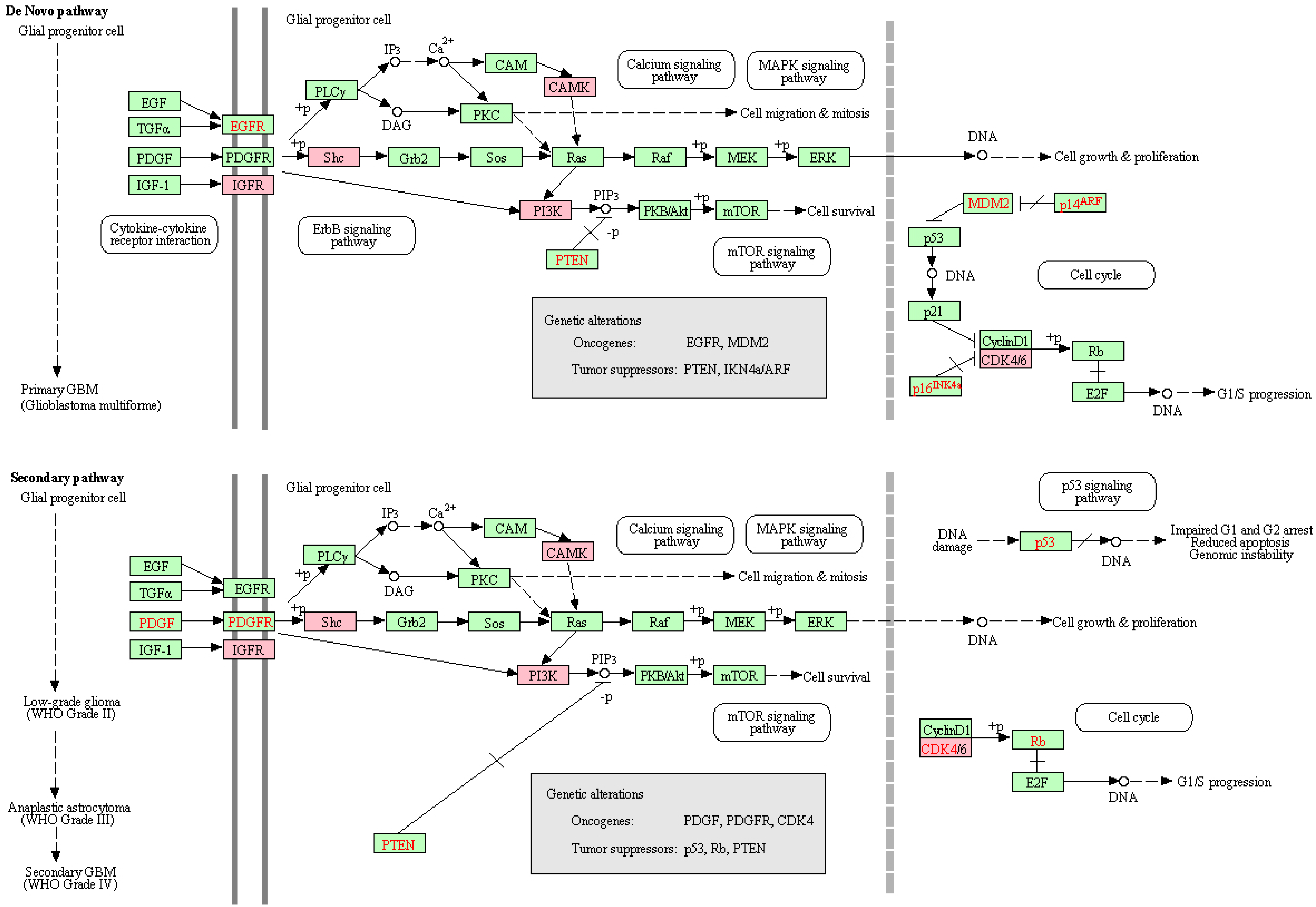
De Novo pathway
Glial progenitor cell
Primary GBM
(Glioblastoma multiforme)
EGF
TGFα
PDGF
IGF-1
EGFR
PDGFR
IGFR
Cytokine-cytokine receptor interaction
Glial progenitor cell
PLCγ
IP3
Ca2+
CAM
CAMK
DAG
PKC
Calcium signaling pathway
MAPK signaling pathway
Cell migration & mitosis
+p
Shc
Grb2
Sos
Ras
Raf
MEK
ERK
DNA
Cell growth & proliferation
ErbB signaling pathway
PI3K
PIP3
-p
PTEN
PKB/Akt
mTOR
Cell survival
mTOR signaling pathway
Genetic alterations
Oncogenes: EGFR, MDM2
Tumor suppressors: PTEN, IKN4a/ARF
MDM2
p14ARF
p53
p21
Cell cycle
CyclinD1
CDK4/6
p16INK4a
Rb
E2F
G1/S progression
Secondary pathway
Glial progenitor cell
Low-grade glioma
(WHO Grade II)
Anaplastic astrocytoma
(WHO Grade III)
Secondary GBM
(WHO Grade IV)
p53 signaling pathway
DNA damage
Impaired G1 and G2 arrest
Reduced apoptosis
Genomic instability
Genetic alterations
Oncogenes: PDGF, PDGFR, CDK4
Tumor suppressors: p53, Rb, PTEN

Bladder Cancer

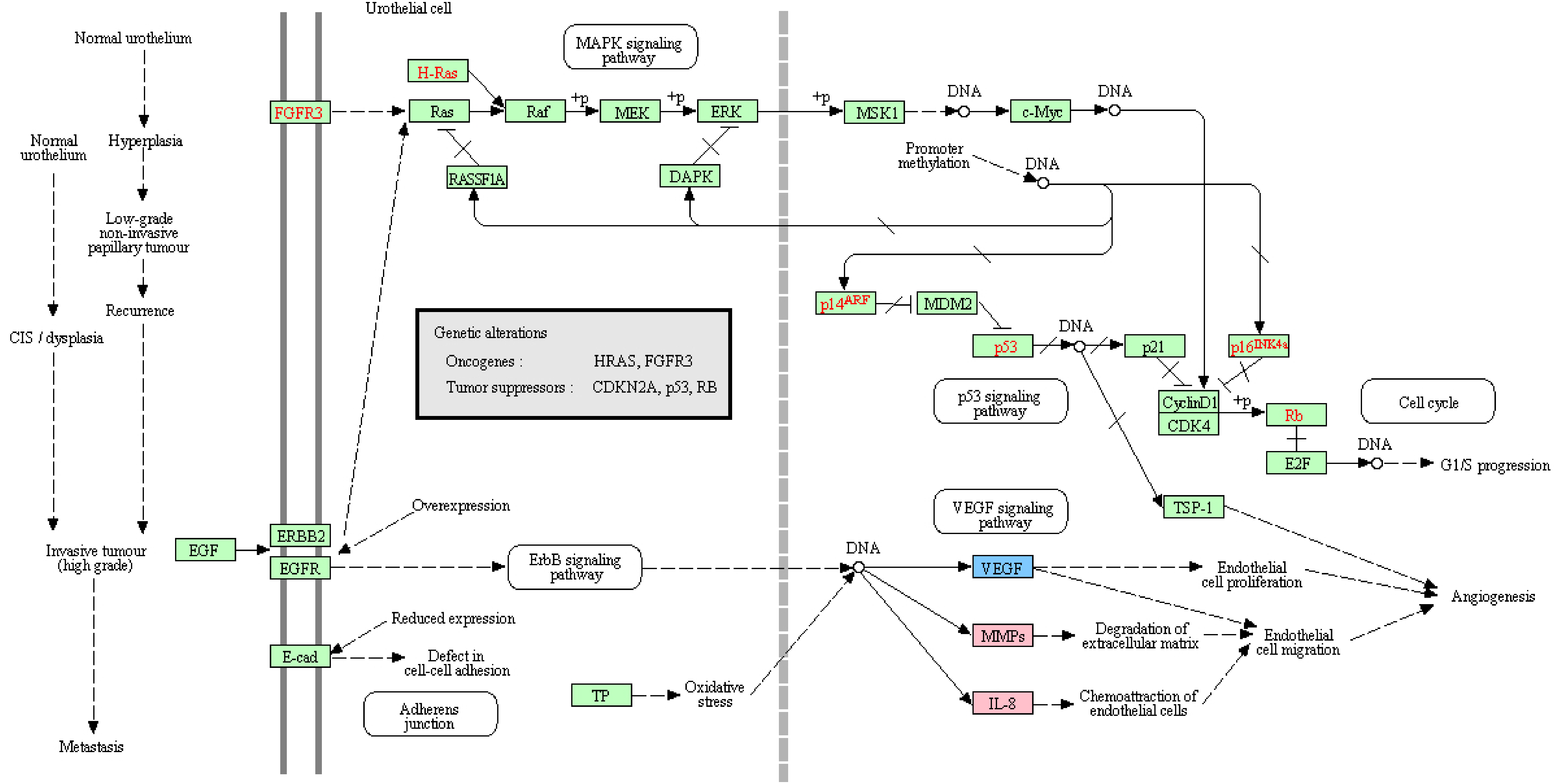

Small Cell Lung Cancer

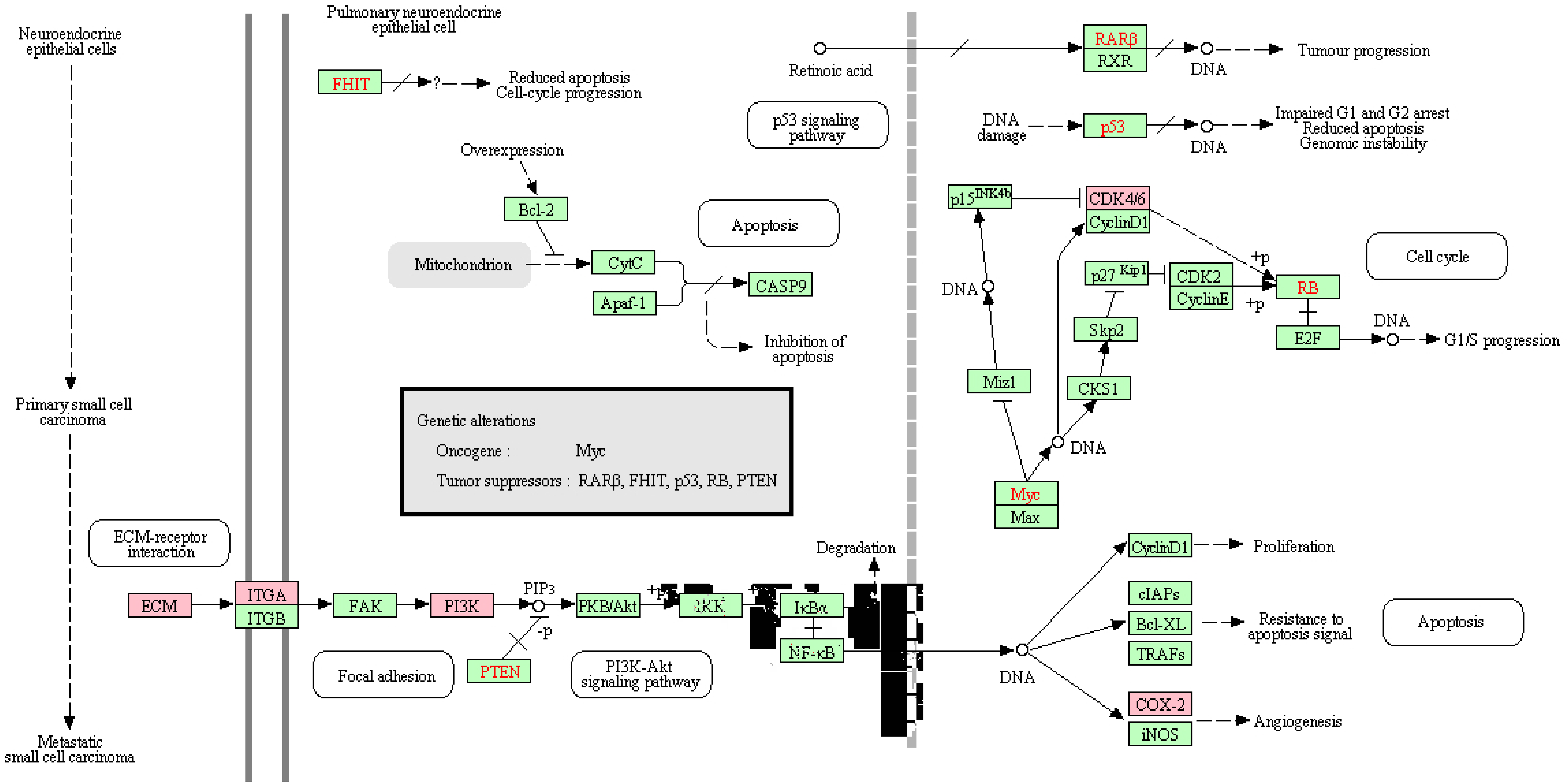

Chronic Myeloid Leukemia

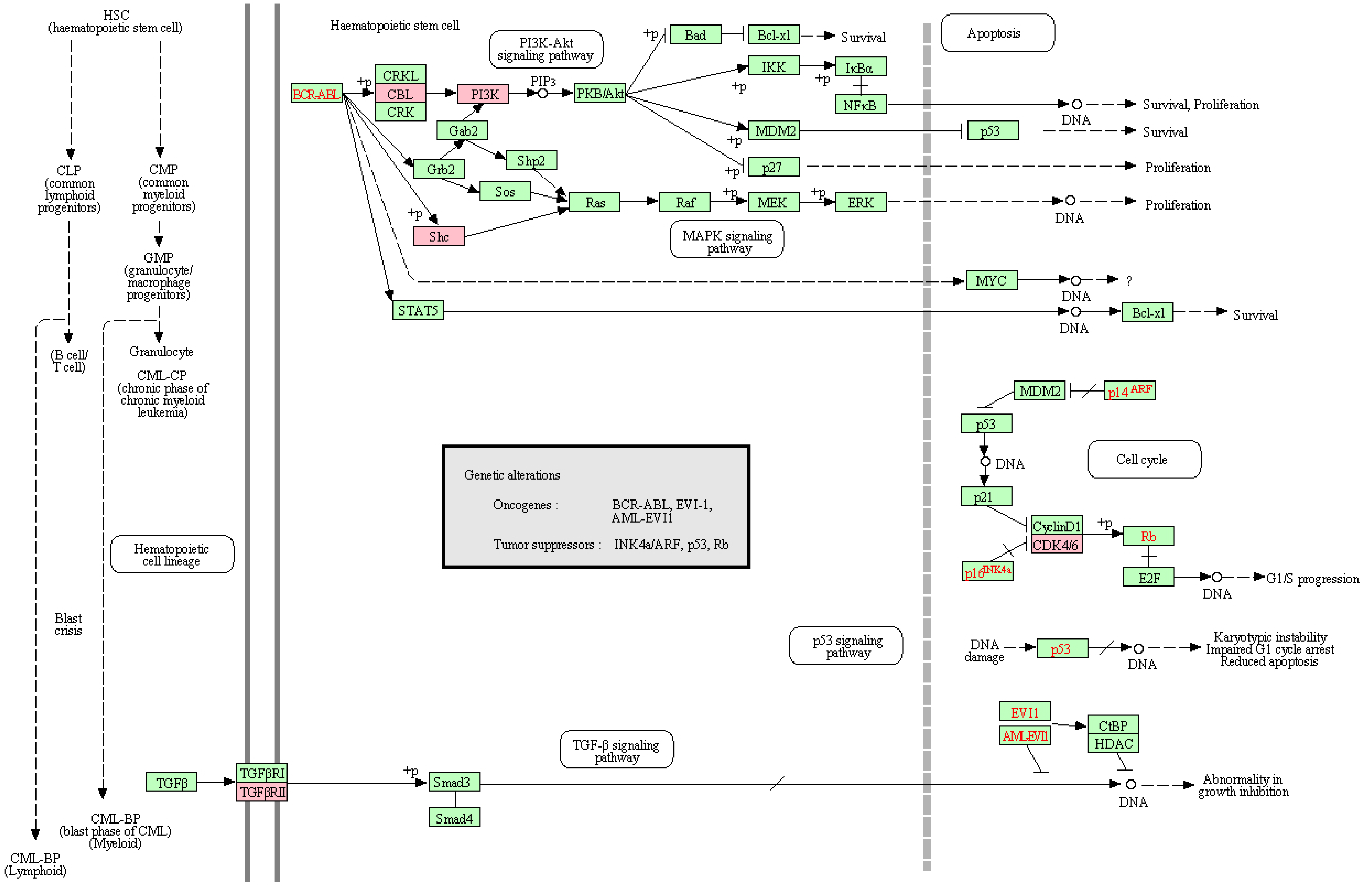

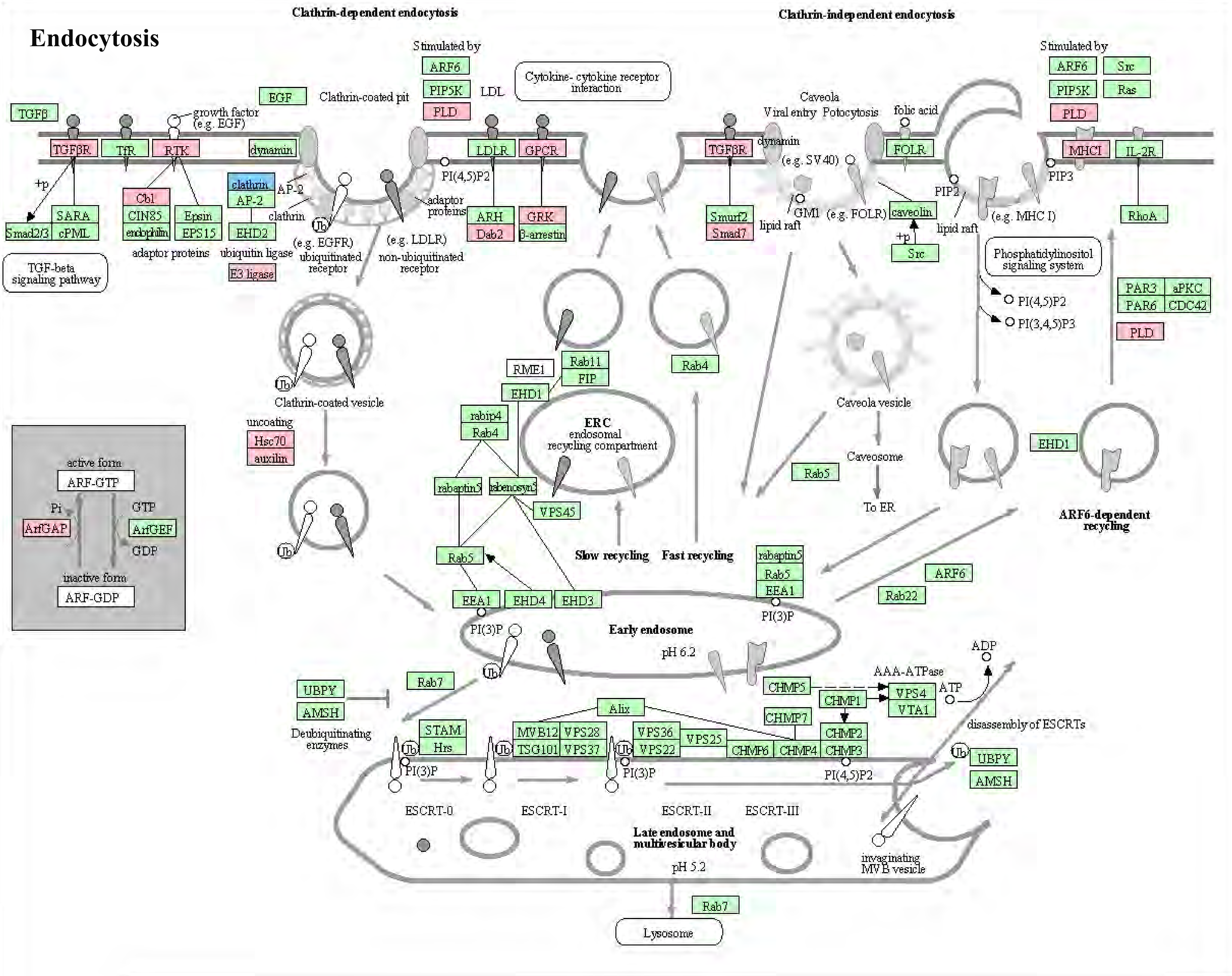

Endocytosis
Clathrin-dependent endocytosis
Clathrin-independent endocytosis
Stimulated by
ARF6
PIP5K
PLD
LDL
Cytokine- cytokine receptor interaction
EGF
Clathrin-coated pit
TGFβ
growth factor (e.g. EGF)
TGFβR
TfR
RTK
dynamin
LDLR
GPCR
PI(4,5)P2
clathrin
AP-2
+p
Cbl
CIN85
endophilin
Epsin
EPS15
EHD2
SARA
Smad2/3
cPML
adaptor proteins
proteins
ubiquitin ligase
E3 ligase
Ub
(e.g. EGFR) ubiquitinated receptor
(e.g. LDLR) non-ubiquitinated receptor
ARH
Dab2
GRK
β-arrestin
TGF-beta signaling pathway
Smurf2
Smad7
Caveola
Viral entry
Potocytosis
folic acid
(e.g. SV40)
GM1
lipid raft
(e.g. FOLR)
FOLR
caveolin
Src
Ras
MHCI
IL-2R
PIP2
PIP3
(e.g. MHC I)
RhoA
Phosphatidylinositol signaling system
PI(4,5)P2
PI(3,4,5)P3
PAR3
aPKC
PAR6
CDC42
Clathrin-coated vesicle
uncoating
Hsc70
auxilin
RME1
Rab11
FIP
EHD1
rabip4
Rab4
ERC endosomal recycling compartment
rabaptin5
rabenosyn5
VPS45
Rab5
EEA1
EHD4
EHD3
Slow recycling
Fast recycling
Caveola vesicle
Caveosome
To ER
ARF6-dependent recycling
Rab22
active form
ARF-GTP
Pi
GTP
ArfGAP
ArfGEF
GDP
inactive form
ARF-GDP
PI(3)P
Early endosome
pH 6.2
AAA-ATPase
ADP
ATP
Rab7
UBPY
AMSH
Deubiquitinating enzymes
CHMP5
VPS4
VTA1
CHMP1
CHMP7
CHMP2
CHMP3
Alix
STAM
Hrs
MVB12
VPS28
TSG101
VPS37
VPS36
VPS22
VPS25
CHMP6
CHMP4
disassembly of ESCRTs
ESCRT-0
ESCRT-I
ESCRT-II
ESCRT-III
Late endosome and multivesicular body
pH 5.2
invaginating MVB vesicle
Lysosome

Osteoclast Differentiation

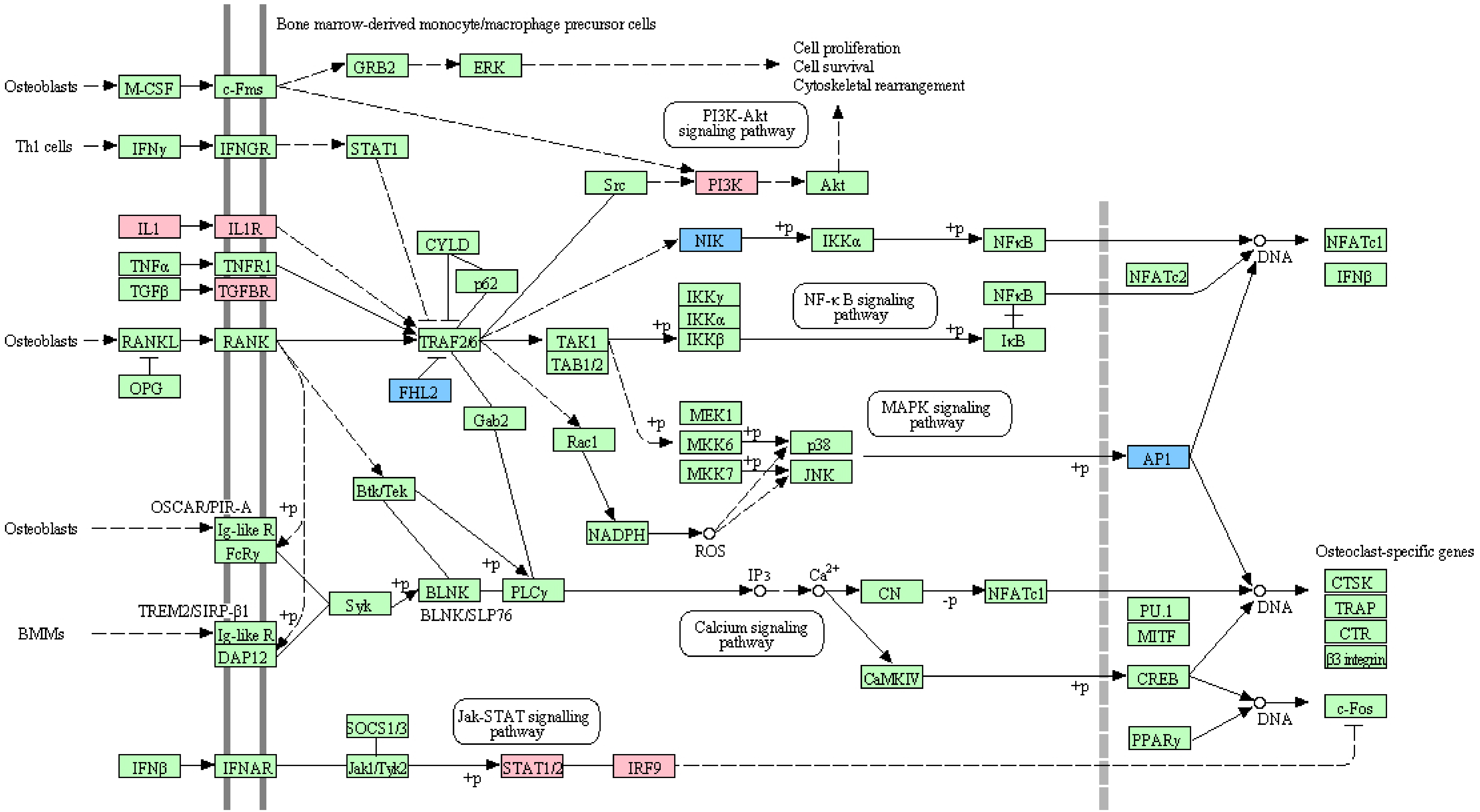

Focal Adhesion

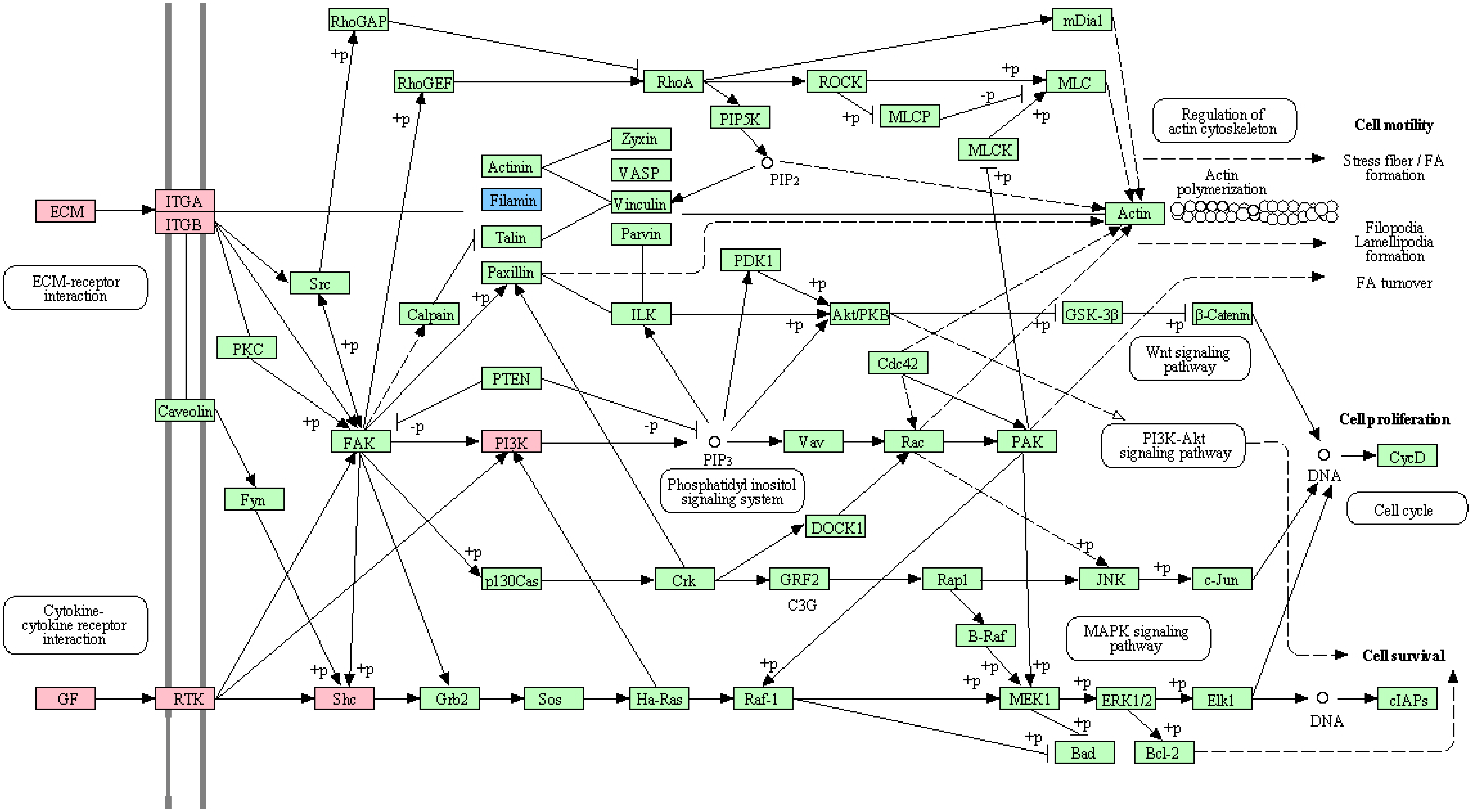

ECM-Receptor Interaction

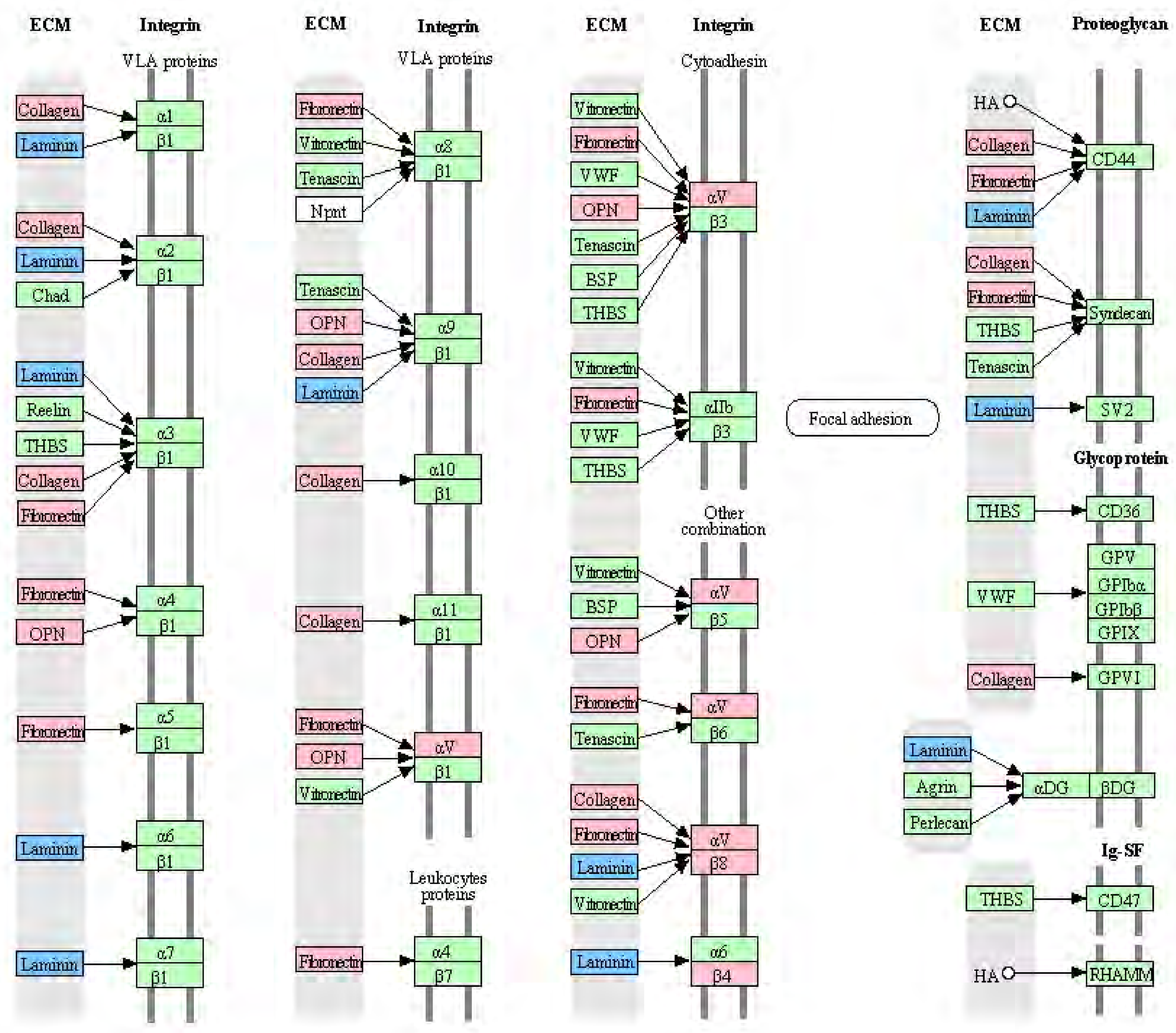

Cell Adhesion Molecules

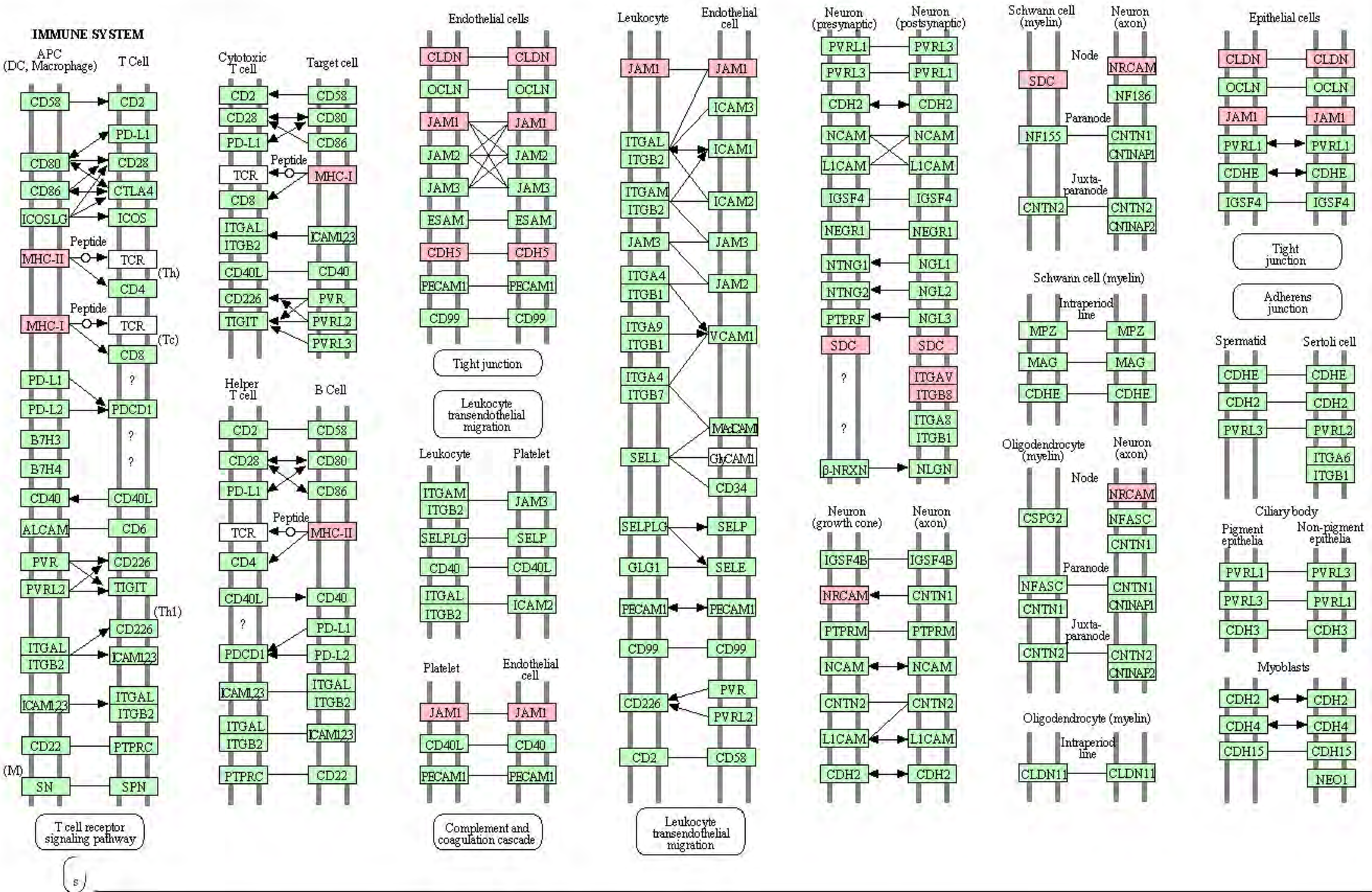

Complement and Coagulation Cascades

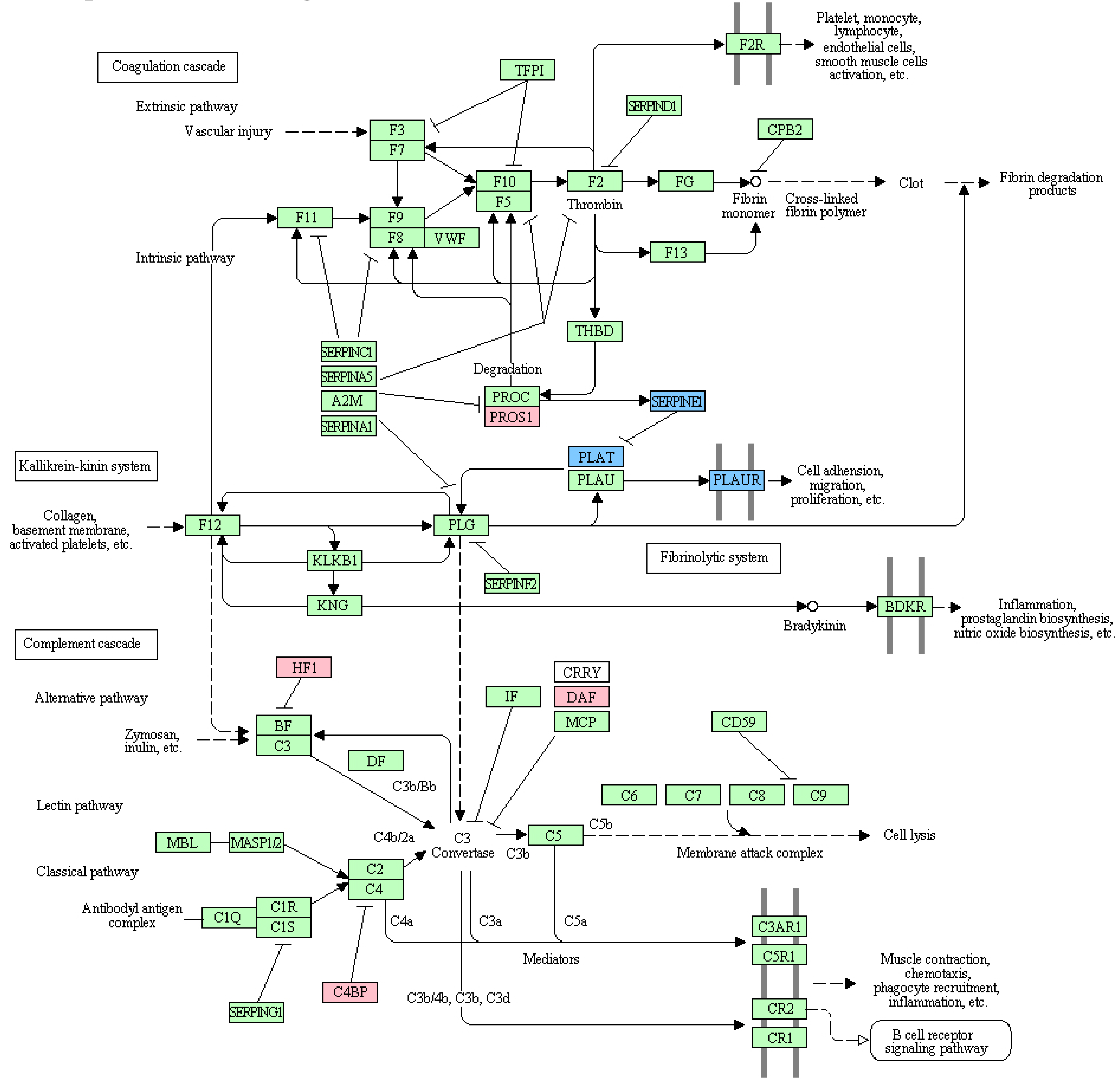

NOD-Like Receptor Signaling Pathway

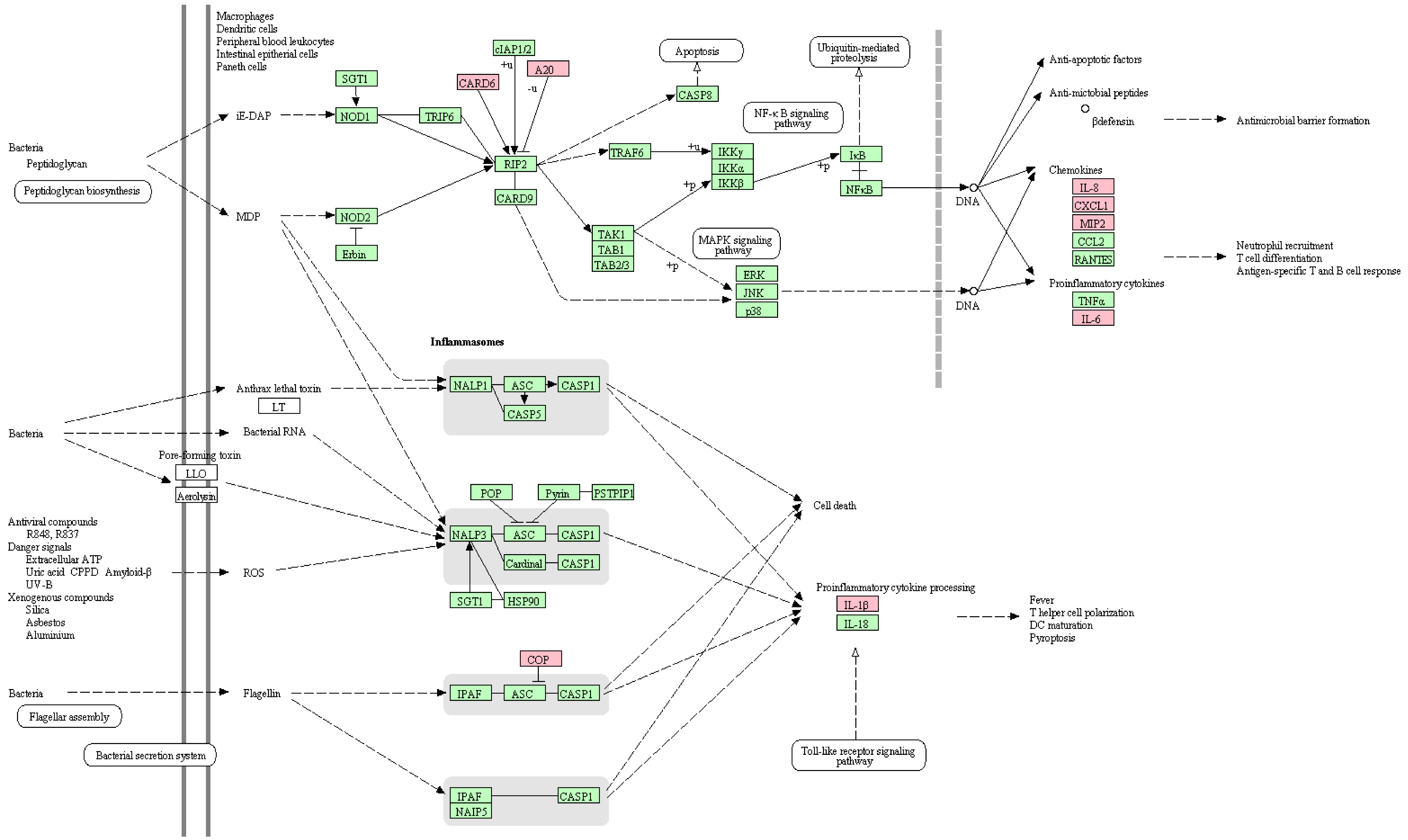

Hematopoietic Cell Lineage

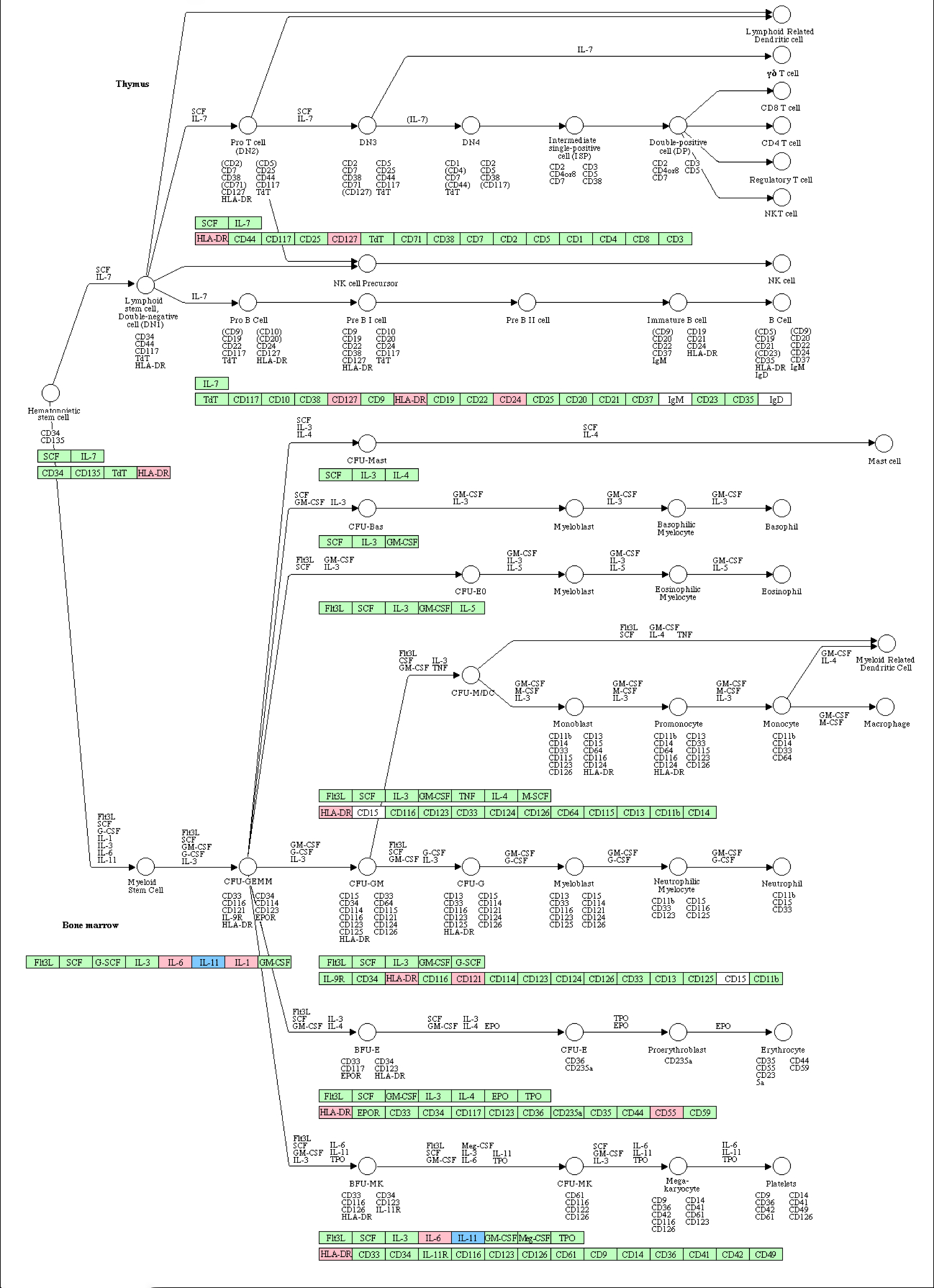

FcγR-Mediated Phagocytosis

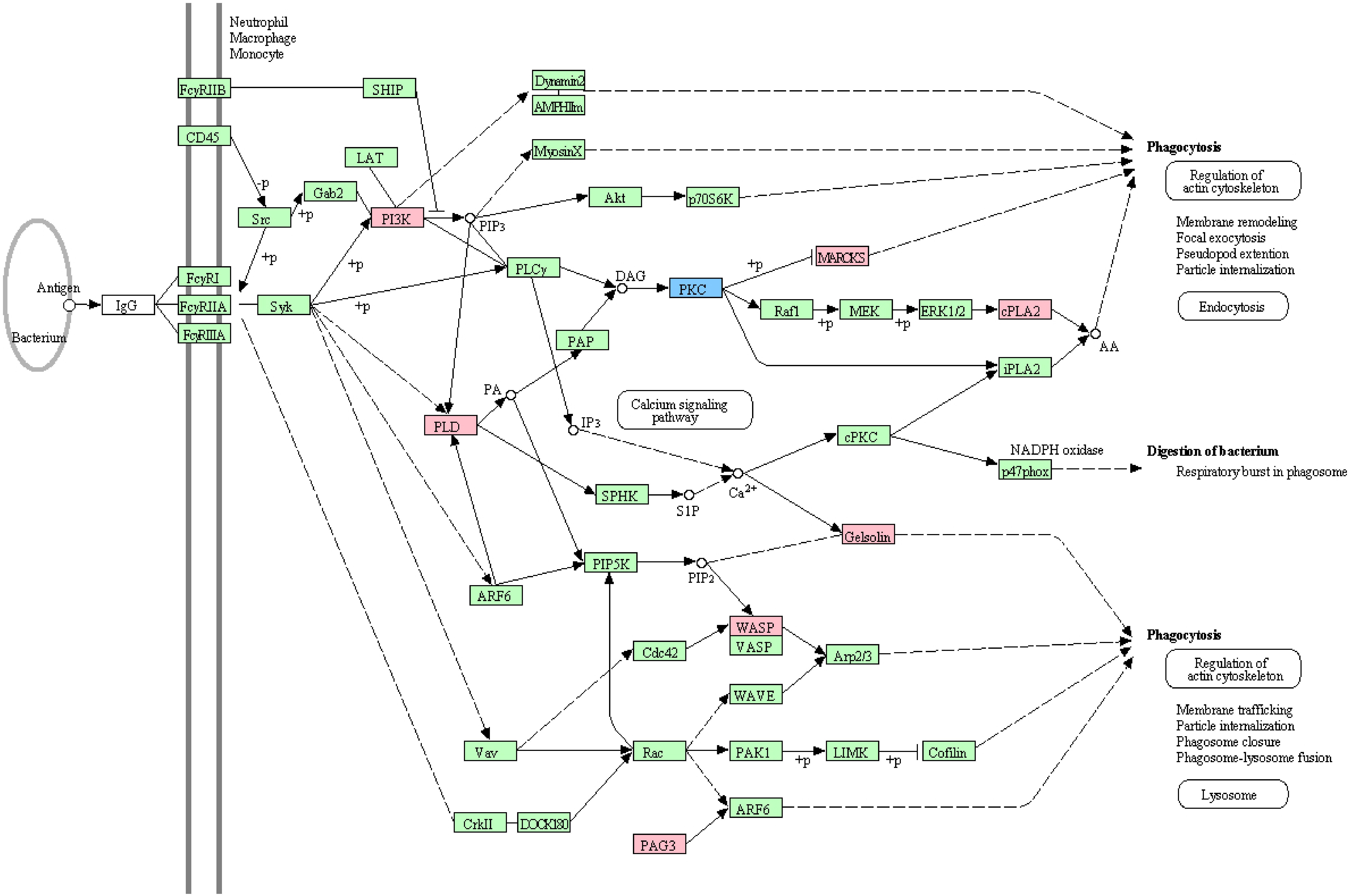

Type I Diabetes Mellitus

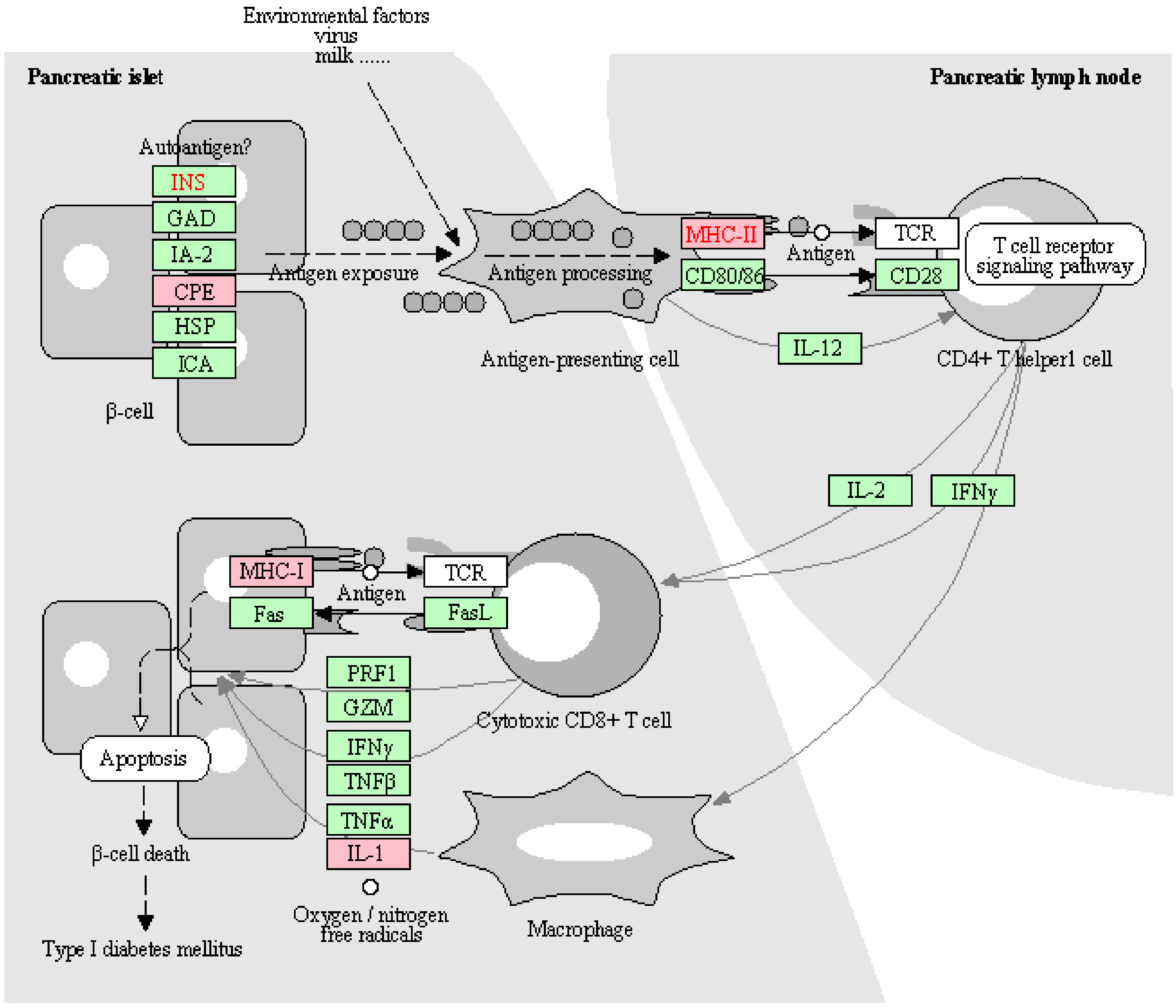

Bacterial Invasion of Epithelial Cells

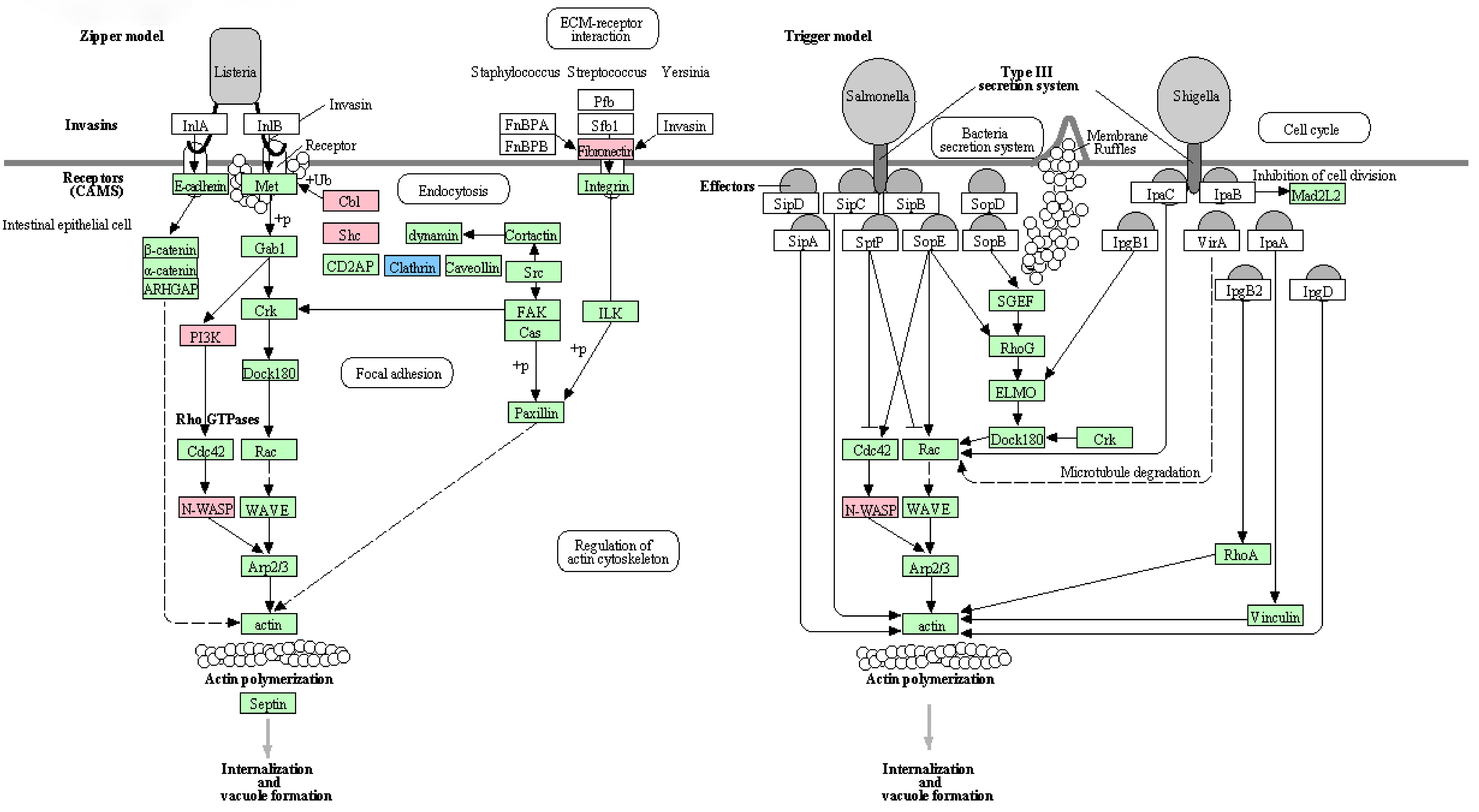

Malaria

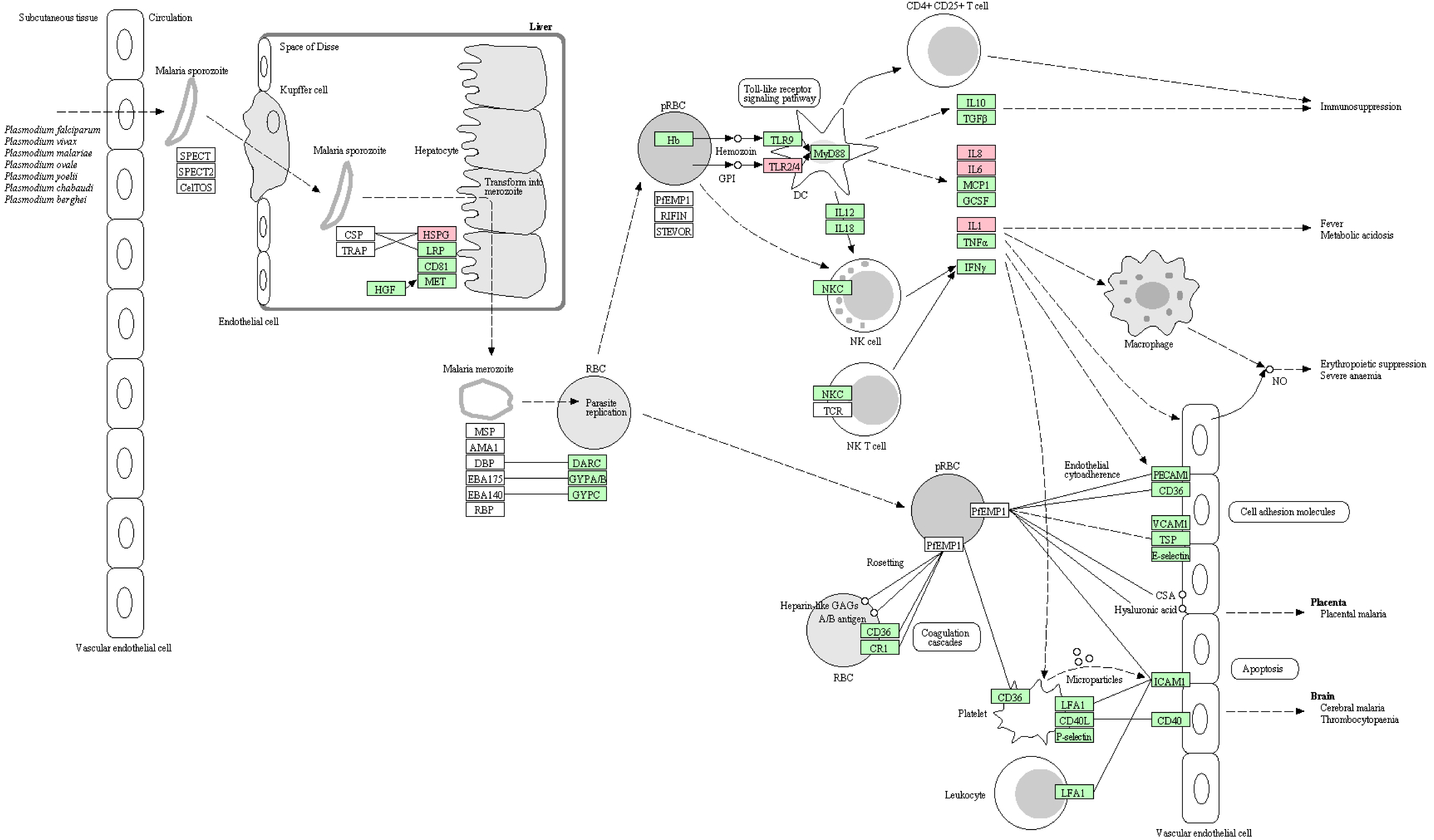

Subcutaneous tissue
Circulation
Malaria sporozoite
Plasmodium falciparum
Plasmodium vivax
Plasmodium malariae
Plasmodium ovale
Plasmodium yoelii
Plasmodium chabaudi
Plasmodium berghei
SPECT
SPECT2
CelTOS
Vascular endothelial cell
Liver
Space of Disse
Kupffer cell
Malaria sporozoite
Hepatocyte
Transform into merozoite
CSP
TRAP
HSPG
LRP
CD81
MET
HGF
Endothelial cell
Malaria merozoite
MSP
AMA1
DBP
EBA175
EBA140
RBP
RBC
Parasite replication
DARC
GYPA/B
GYPC
pRBC
Hb
PfEMP1
RIFIN
STEVOR
Hemozoin
GPI
TLR9
TLR2/4
Toll-like receptor signaling pathway
MyD88
DC
IL12
IL18
CD4+ CD25+ T cell
IL10
TGFβ
Immunosuppression
IL8
IL6
MCP1
GCSF
IL1
TNFα
IFNγ
Fever
Metabolic acidosis
NKC
NK cell
NKC
TCR
NK T cell
Macrophage
NO
Erythropoietic suppression
Severe anaemia
pRBC
PfEMP1
PfEMP1
Endothelial cytoadherence
PECAM1
CD36
VCAM1
TSP
E-selectin
Cell adhesion molecules
CSA
Hyaluronic acid
Placenta
Placental malaria
Rosetting
Heparin-like GAGs
A/B antigen
CD36
CR1
RBC
Coagulation cascades
Microparticles
ICAM1
Apoptosis
CD36
Platelet
LFA1
CD40L
P-selectin
CD40
Brain
Cerebral malaria
Thrombocytopaenia
Leukocyte
LFA1
Vascular endothelial cell

Amoebiasis

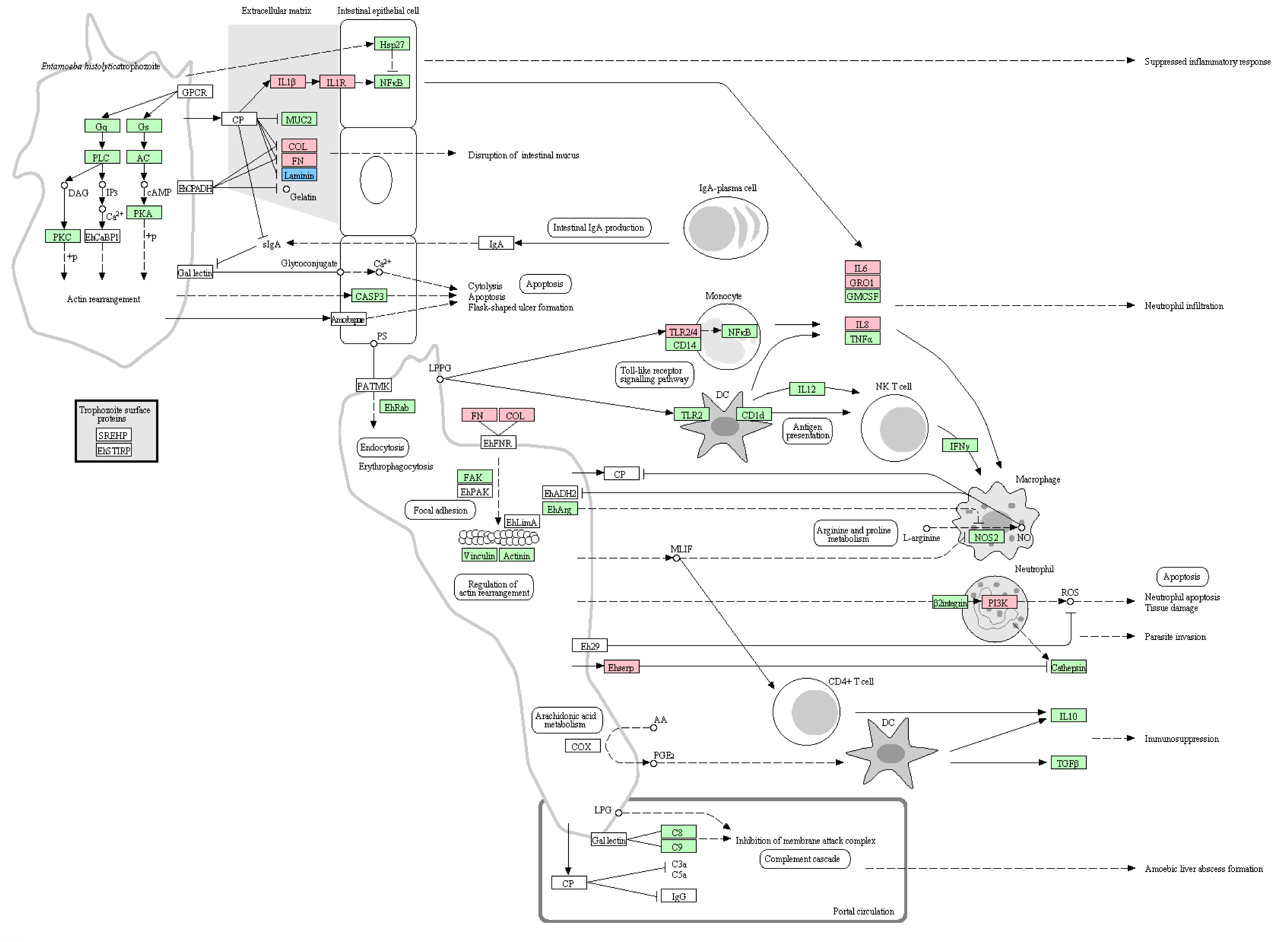

Extracellular matrix
Intestinal epithelial cell
Entamoeba histolytica trophozoite
Hsp27
IL1β
IL1R
NFκB
GPCR
Gq
Gs
PLC
AC
CP
MUC2
COL
FN
Laminin
Gelatin
EhCPADH
DAG
IP3
cAMP
Ca2+
PKA
PKC
EhCaBP1
+p
sIgA
Gal lectin
Glycoconjugate
CASP3
Actin rearrangement
Amoebapore
PS
Cytolysis
Apoptosis
Flask-shaped ulcer formation
Disruption of intestinal mucus
Suppressed inflammatory response
IgA-plasma cell
Intestinal IgA production
IgA
IL6
GRO1
GMCSF
IL8
TNFα
Neutrophil infiltration
Monocyte
TLR2/4
CD14
Toll-like receptor signalling pathway
LPPG
PATMK
EhRab
Endocytosis
Erythrophagocytosis
Trophozoite surface proteins
SREHP
EhSTIRP
DC
TLR2
CD1d
IL12
NK T cell
Antigen presentation
IFNγ
FN
COL
EhFNR
FAK
EhPAK
Focal adhesion
EhLimA
Vinculin
Actinin
Regulation of actin rearrangement
CP
EhADH2
EhArg
Macrophage
Arginine and proline metabolism
L-arginine
NOS2
NO
MLIF
Neutrophil
β2integrin
PI3K
ROS
Apoptosis
Neutrophil apoptosis
Tissue damage
Parasite invasion
Eh29
Ehserp
Cathepsin
CD4+ T cell
IL10
TGFβ
Immunosuppression
Arachidonic acid metabolism
AA
COX
PGE2
LPG
Gal lectin
C8
C9
Inhibition of membrane attack complex
Complement cascade
C3a
C5a
IgG
CP
Amoebic liver abscess formation
Portal circulation

Measles

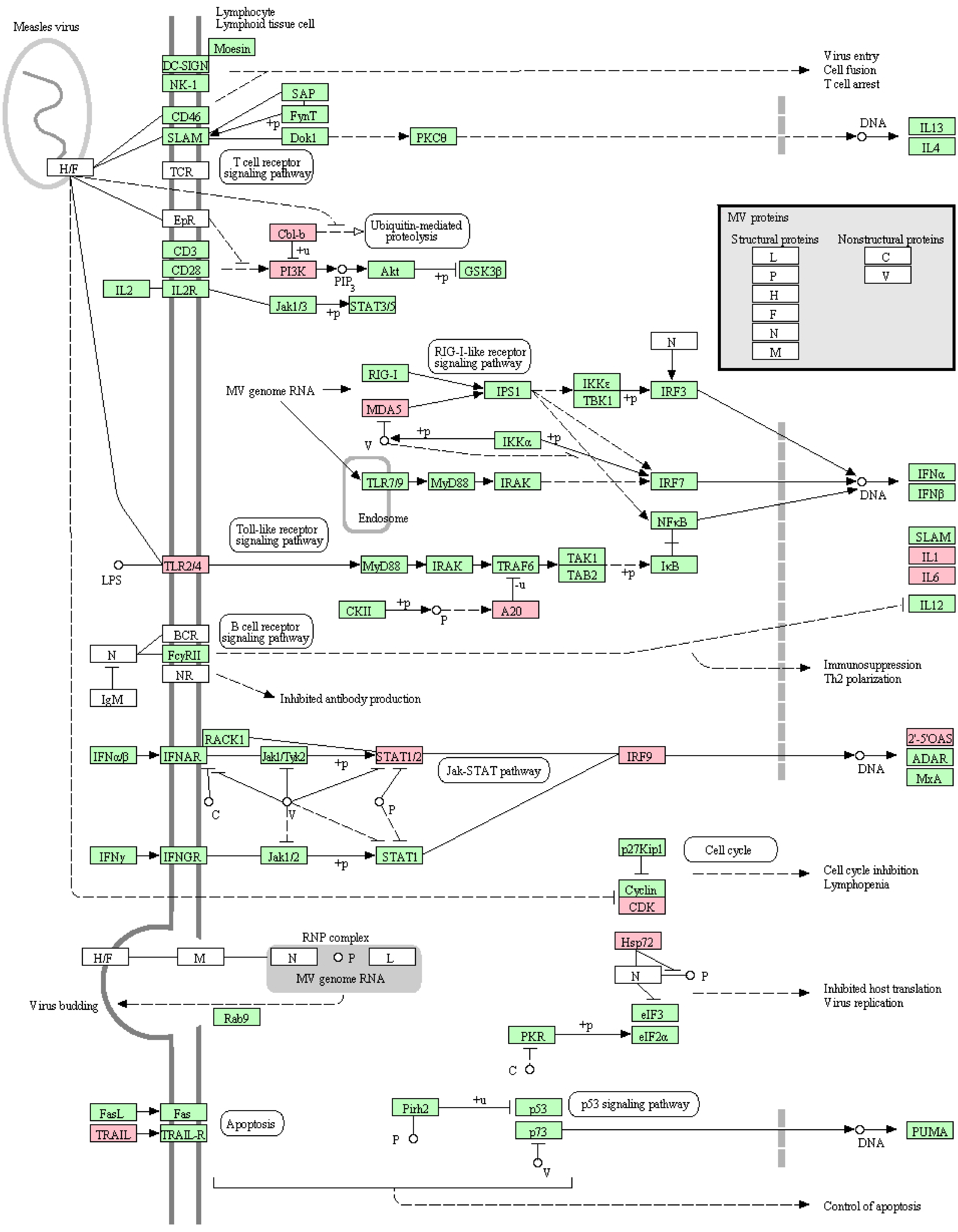

Measles virus
Lymphocyte
Lymphoid tissue cell
Moesin
DC-SIGN
NK-1
CD46
SLAM
TCR
H/F
SAP
FynT
Dok1
PKCθ
T cell receptor signaling pathway
Virus entry
Cell fusion
T cell arrest
DNA
IL13
IL4
EpR
Cbl-b
Ubiquitin-mediated proteolysis
CD3
CD28
PI3K
PIP3
Akt
GSK3β
IL2
IL2R
Jak1/3
STAT3/5
MV proteins
Structural proteins
Nonstructural proteins
L
P
H
F
N
M
C
V
RIG-I-like receptor signaling pathway
RIG-I
MDA5
MV genome RNA
IPS1
IKKε
TBK1
IRF3
IKKα
TLR7/9
MyD88
IRAK
IRF7
Endosome
NFκB
IFNα
IFNβ
Toll-like receptor signaling pathway
LPS
TLR2/4
TRAF6
TAK1
TAB2
IκB
CKII
A20
SLAM
IL1
IL6
IL12
B cell receptor signaling pathway
BCR
FcγRII
NR
IgM
Inhibited antibody production
Immunosuppression
Th2 polarization
RACK1
IFNα/β
IFNAR
Jak1/Tyk2
STAT1/2
Jak-STAT pathway
IRF9
2'-5'OAS
ADAR
MxA
IFNγ
IFNGR
Jak1/2
STAT1
p27Kip1
Cell cycle
Cell cycle inhibition
Lymphopenia
Cyclin
CDK
RNP complex
Hsp72
Virus budding
Rab9
Inhibited host translation
Virus replication
eIF3
PKR
eIF2α
FasL
Fas
TRAIL
TRAIL-R
Apoptosis
Pirh2
p53
p53 signaling pathway
p73
PUMA
Control of apoptosis

Influenza A

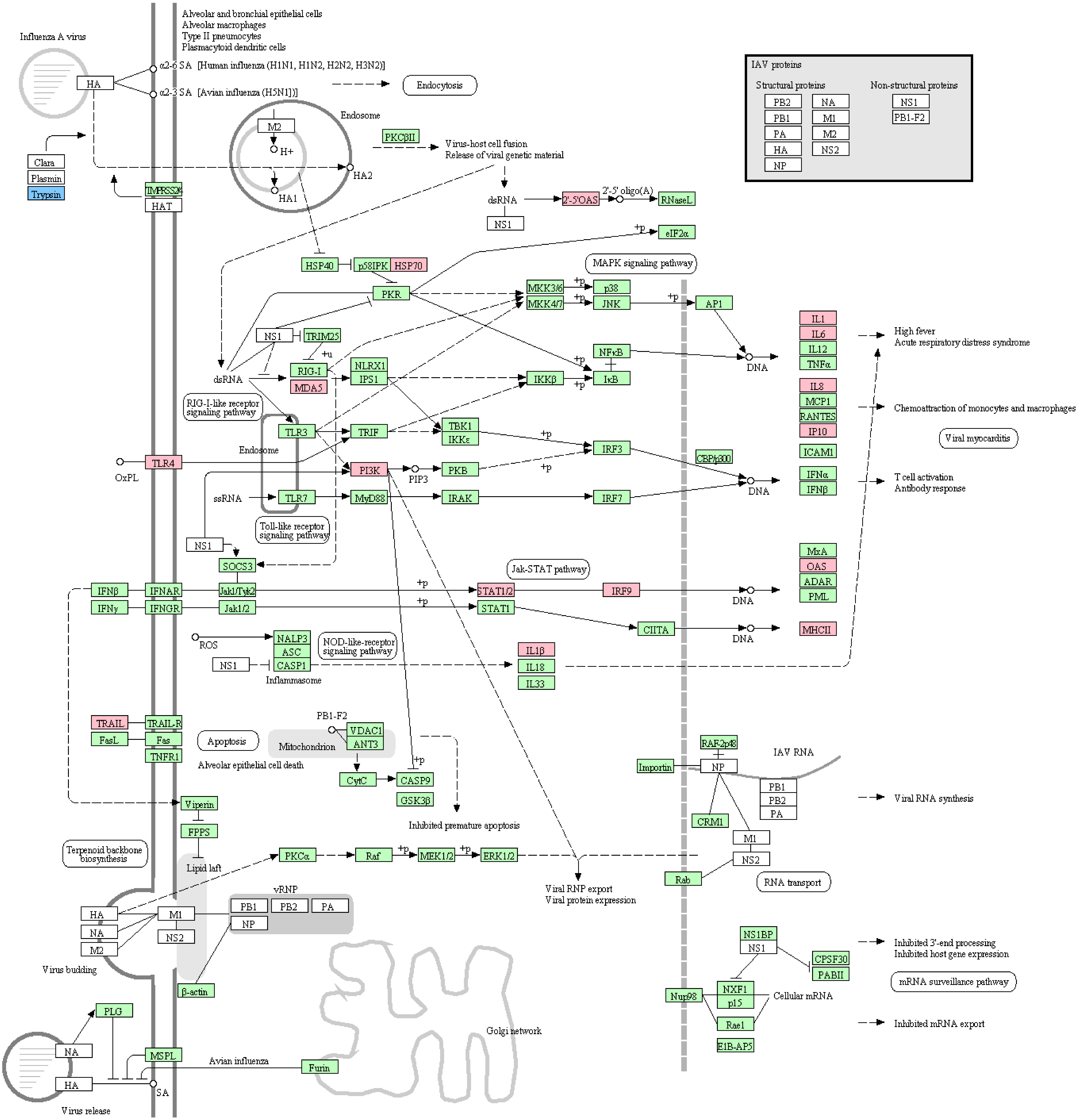

Alveolar and bronchial epithelial cells
Alveolar macrophages
Type II pneumocytes
Plasmacytoid dendritic cells
Influenza A virus
HA
α2-6 SA [Human influenza (H1N1, H1N2, H2N2, H3N2)]
α2-3 SA [Avian influenza (H5N1)]
Endocytosis
Endosome
M2
H+
HA2
HA1
Clara
Plasmin
Trypsin
TMPRSS2/4
HAT
PKCβII
Virus-host cell fusion
Release of viral genetic material
dsRNA
2'-5'OAS
2'-5' oligo(A)
RNaseL
NS1
eIF2α
+p
IAV proteins
Structural proteins
Non-structural proteins
PB2
PB1
PA
HA
NP
NA
M1
M2
NS2
NS1
PB1-F2
HSP40
p58IPK
HSP70
PKR
MAPK signaling pathway
MKK3/6
MKK4/7
p38
JNK
AP1
NS1
TRIM25
+u
RIG-I
MDA5
NLRX1
IPS1
IKKβ
NFκB
IκB
DNA
IL1
IL6
IL12
TNFα
High fever
Acute respiratory distress syndrome
RIG-I-like receptor signaling pathway
TLR3
TRIF
TBK1
IKKε
IRF3
IL8
MCP1
RANTES
IP10
ICAM1
Chemoattraction of monocytes and macrophages
Viral myocarditis
Endosome
OxPL
TLR4
PI3K
PIP3
PKB
CBP/p300
IFNα
IFNβ
T cell activation
Antibody response
ssRNA
TLR7
MyD88
IRAK
IRF7
Toll-like receptor signaling pathway
NS1
SOCS3
Jak-STAT pathway
IFNβ
IFNAR
Jak1/Tyk2
STAT1/2
IRF9
IFNγ
IFNGR
Jak1/2
STAT1
CIITA
MxA
OAS
ADAR
PML
MHCII
ROS
NALP3
ASC
CASP1
NOD-like-receptor signaling pathway
Inflammasome
IL1β
IL18
IL33
TRAIL
TRAIL-R
FasL
Fas
TNFR1
Apoptosis
Mitochondrion
Alveolar epithelial cell death
PB1-F2
VDAC1
ANT3
CytC
CASP9
GSK3β
Inhibited premature apoptosis
RAF-2p48
Importin
NP
IAV RNA
PB1
PB2
PA
CRM1
M1
NS2
Rab
RNA transport
Viral RNA synthesis
Viperin
FPPS
Terpenoid backbone biosynthesis
Lipid laft
PKCα
Raf
MEK1/2
ERK1/2
Viral RNP export
Viral protein expression
HA
NA
M2
M1
NS2
vRNP
PB1
PB2
PA
NP
Virus budding
β-actin
NS1BP
NS1
CPSF30
PABII
Inhibited 3'-end processing
Inhibited host gene expression
mRNA surveillance pathway
Nup98
NXF1
p15
Rae1
E1B-AP5
Cellular mRNA
Inhibited mRNA export
PLG
NA
HA
MSPL
Furin
Avian influenza
SA
Golgi network
Virus release

Rheumatoid Arthritis

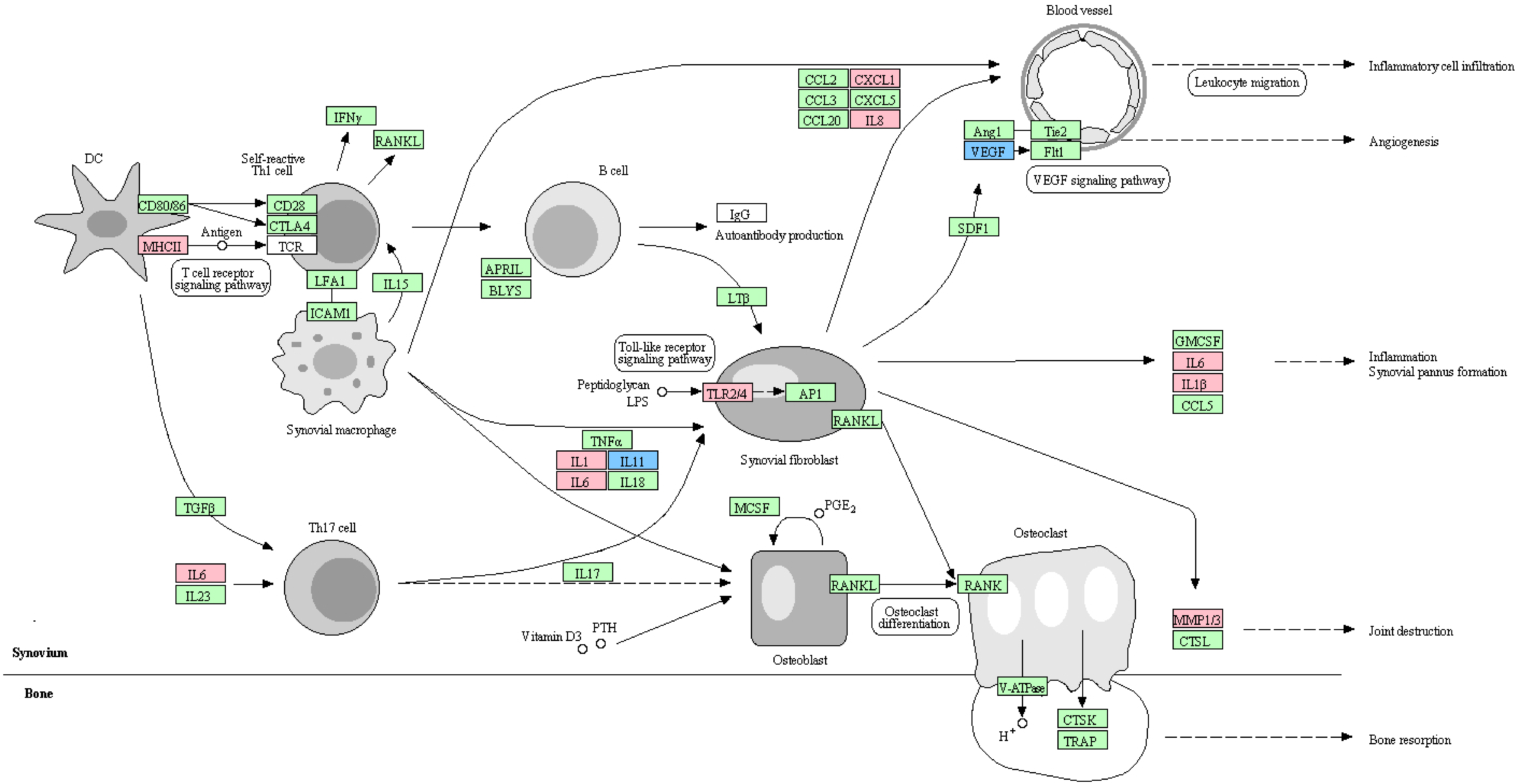

Supplement: Supplementary file 1 [file ijms-19-01451-s001.zip › Supplementary Figure 1.pdf]
